# Structure and expression of the maize (*Zea mays *L.) SUN-domain protein gene family: evidence for the existence of two divergent classes of SUN proteins in plants

**DOI:** 10.1186/1471-2229-10-269

**Published:** 2010-12-08

**Authors:** Shaun P Murphy, Carl R Simmons, Hank W Bass

**Affiliations:** 1 Institute of Molecular Biophysics, The Florida State University, Tallahassee, FL, USA 32306-4370; 2 Pioneer Hi-Bred International, Johnston, IA, USA 50131; 3 Department of Biological Science, The Florida State University, Tallahassee, FL, USA 32306-4370

## Abstract

**Background:**

The nuclear envelope that separates the contents of the nucleus from the cytoplasm provides a surface for chromatin attachment and organization of the cortical nucleoplasm. Proteins associated with it have been well characterized in many eukaryotes but not in plants. SUN (Sad1p/Unc-84) domain proteins reside in the inner nuclear membrane and function with other proteins to form a physical link between the nucleoskeleton and the cytoskeleton. These bridges transfer forces across the nuclear envelope and are increasingly recognized to play roles in nuclear positioning, nuclear migration, cell cycle-dependent breakdown and reformation of the nuclear envelope, telomere-led nuclear reorganization during meiosis, and karyogamy.

**Results:**

We found and characterized a family of maize SUN-domain proteins, starting with a screen of maize genomic sequence data. We characterized five different maize *ZmSUN *genes *(ZmSUN1-5)*, which fell into two classes (probably of ancient origin, as they are also found in other monocots, eudicots, and even mosses). The first (*ZmSUN1*, *2*), here designated canonical C-terminal SUN-domain (CCSD), includes structural homologs of the animal and fungal SUN-domain protein genes. The second (*ZmSUN3, 4, 5*), here designated plant-prevalent mid-SUN 3 transmembrane (PM3), includes a novel but conserved structural variant SUN-domain protein gene class. Mircroarray-based expression analyses revealed an intriguing pollen-preferred expression for *ZmSUN5 *mRNA but low-level expression (50-200 parts per ten million) in multiple tissues for all the others. Cloning and characterization of a full-length cDNA for a PM3-type maize gene, *ZmSUN4*, is described. Peptide antibodies to ZmSUN3, 4 were used in western-blot and cell-staining assays to show that they are expressed and show concentrated staining at the nuclear periphery.

**Conclusions:**

The maize genome encodes and expresses at least five different SUN-domain proteins, of which the PM3 subfamily may represent a novel class of proteins with possible new and intriguing roles within the plant nuclear envelope. Expression levels for *ZmSUN*1-4 are consistent with basic cellular functions, whereas *ZmSUN*5 expression levels indicate a role in pollen. Models for possible topological arrangements of the CCSD-type and PM3-type SUN-domain proteins are presented.

## Background

### Organization of Chromatin and the Nuclear Envelope in Animals and Plants

Genomic DNA is packaged by proteins into chromatin that resides within the nuclear space in eukaryotic organisms. Within this three-dimensional space, interphase chromosomes are often observed to occupy discrete, nonoverlapping territories [1,2]. The architecture of the cell nucleus as a whole, in combination with chromatin dynamics, provides a basis for cells' regulation of their gene expression, DNA replication, and DNA repair [2-4]. The eukaryotic cell nucleus is surrounded by a double membrane, the nuclear envelope (NE), which is composed of the inner and outer nuclear membranes, separated by an ~30-nm perinuclear space. The two are connected through nuclear pore complexes, and the space between them is continuous with the lumen of the endoplasmic reticulum (ER). Intrinsic membrane proteins associated with the inner and outer membranes make the NE a rather dynamic membrane system with a multitude of essential functions, including nuclear migration and positioning, cell cycle-dependent NE breakdown and reformation, cytoplasmic-nuclear shuttling, calcium signaling, gene expression, genome stability, meiotic chromosome behavior, and karyogamy [3-11]. Mutations in NE-associated proteins, such as nuclear lamins, give rise to a variety of heritable diseases in animals, collectively termed laminopathies, including muscular dystrophy, lipodystrophy, diabetes, dysplasia, leukodystrophy, and progeria [12-16].

Recent advances in yeast and animal NE research have identified SUN (Sad1p/Unc-84) domain homology proteins as key residents of the NE, and their presence in plants is just beginning to be recognized and characterized [17-19]. Despite the conservation of NE-mediated functions between plants and animals and the importance of the NE in plant biology, knowledge of the plant NE proteome remains limited [20-23].

### SUN-Domain Proteins Are Highly Conserved

SUN-domain proteins have gained attention as a family of widely conserved NE-associated proteins that can transmit forces between the nucleus and cytoplasmic motility systems. SUN-domain proteins were first characterized in *Schizosaccharomyces pombe *and *Caenorhabditis elegans *as NE-associated proteins associated with spindle pole-body and nuclear-migration defects, respectively [24,25]. Since then, their analysis in other eukaryotes has extended their functions to roles in chromosome tethering, telomere maintenance, meiotic chromosome behavior, nuclear pore distribution, mitotic chromosome decondensation, and the regulation of apoptosis [13,26-35]. Furthermore, genetic analysis revealed that knockouts within the mouse *SUN1 *gene disrupted the expression of piRNAs and caused a misregulation of a large number of meiosis-specific reproductive genes [36].

In animals and fungi, SUN proteins interact through their C-terminal SUN domains in the perinuclear space with outer-nuclear-membrane-associated KASH (Klarsicht/ANC-1/Syne-1 homology) proteins as part of the LINC (Linker of Nucleoskeleton and Cytoskeleton) complex [13,37-43]. The other members of the KASH-domain family are proteins with cytoplasmic domains and nuclear lamins that reside in the nucleoplasm and therefore allow forces produced within the cytoplasm to be transmitted to the nuclear periphery. Evidence for the expression and NE localization of plant SUN-domain proteins has emerged from studies looking at cytokinesis in *Arabidopsis *and nuclear proteomics in rice [17-19]. Additional studies with AtSUN1 and AtSUN2 firmly establish that these proteins reside in the NE like their animal and fungal counterparts [17-19].

### SUN-Domain Proteins and Meiotic Chromosome Behavior

Some animal and fungal SUN-domain proteins are known to have a conserved role in meiotic chromosome behavior [9,13,33,34,44]. During meiotic prophase I, a dramatic reorganization of the nucleus occurs in which the chromosomes compact and telomeres attach themselves to the NE by an unknown active mechanism, cluster into a bouquet arrangement, and finally disperse along the surface of the inner nuclear membrane. The formation and dynamics of the bouquet configuration of meiotic chromosomes contribute to proper homologous chromosome pairing, synapsis, recombination, and segregation [45-50].

In maize, meiotic telomere clustering has been demonstrated to occur *de novo *on the NE during meiotic prophase I, and the temporal patterns are nearly identical to those in mammals [45,51]. Interestingly, genetic disruption of the *SUN1 *gene in mouse leads to defects in meiotic telomere-NE association, pairing, synapsis, and recombination, a phenotype remarkably similar to those of two maize synapsis-deficient mutants, *desynaptic *(*dy*) and *desynaptic1 *(*dsy1*) [33,52].

We set out to identify maize *SUN *genes to provide a foundation for analysis of meiosis and other nuclear processes in plants. Using bioinformatics and molecular approaches, we discovered five different *SUN*-domain genes (here designated *ZmSUN1*-*5*) in the maize genome. We present evidence that these fall into two subfamilies, which we call canonical C-terminal SUN domain (CCSD) and plant-prevalent mid-SUN 3 transmembrane (PM3). We also provide the first evidence for expression and localization of PM3-type proteins and discuss the possible significance of this novel structural-variant subfamily.

## Results and Discussion

### Identification of Maize Genes Encoding Canonical C-terminal SUN-Domain (CCSD) Proteins

A reference genome sequence was recently produced for the inbred line B73 (B73 RefGen_v1 [53]). The *SUN *genes described here refer to B73 sequences where possible, although many of the public cDNA and EST sequences in GenBank are from multiple other inbred lines of maize. We identified SUN-domain protein genes in a model plant genetic system by using a BLAST homology search of the maize genome queried with a fungal SUN-domain protein Sad1p, from *S. pombe *[24]. The two different putative maize SUN-domain protein genes we initially identified, *ZmSUN1 *and *ZmSUN2*, were each predicted to encode ~ 50-kDa proteins. When the predicted protein sequences were used to query the Conserved Domain Database (version 2.21, NCBI), each revealed the presence of a single conserved domain, the SUN/Sad1_UNC superfamily (pfam07738), near the C-terminus of the proteins. These maize genes are homologous to recently characterized plant SUN-domain protein genes from *Arabidopsis *(*AtSUN1*, *AtSUN2 *[54,55]) and rice (*OsSad1 *[18]). Experimental evidence from heterologous expression assays with fluorescent protein fusions indicates that these *Arabidopsis *and rice CCSD proteins are localized at the NE. The presence of a C-terminal SUN domain and the NE localization are among the defining features of animal and fungal SUN proteins [9,13,38]. Plant genomes therefore appear to encode canonical C-terminal SUN-domain (CCSD) type proteins, an observation that is not surprising given the conserved role of these proteins in basic eukaryotic processes such as meiosis, mitosis, and nuclear positioning [8,9,38,39,42].

### Discovery of Maize Genes Encoding PM3-type of SUN-domain Proteins

Additional bioinformatic analyses revealed that the maize genome encodes not only CCSD-type SUN-domain proteins but also a unique family of SUN-domain protein genes not previously described. Members of this second group of genes (*ZmSUN3*, *ZmSUN4*, and *ZmSUN5*) encode slightly larger proteins with three transmembrane domains, a single SUN-domain that is not at the C-terminus but rather in the middle of the protein, and a highly-conserved domain of unknown function that we refer to as the PM3-associated domain (PAD). When used to query the Conserved Domain Database, these predicted proteins also revealed the presence of the SUN/Sad1_UNC superfamily, pfam07738. Homologous protein sequences with similar secondary structure and motif arrangement were found to be prevalent within plant genomes. We refer to this group, therefore, as the PM3-type (Plant-prevalent Mid-SUN 3 transmembrane) SUN-domain proteins, as represented by the founding members *ZmSUN3*, *ZmSUN4*, and *ZmSUN5*. A summary of the five maize SUN-domain protein genes is provided in Table 1 and the properties and motifs of the CCSD and PM3 subfamilies of these proteins are summarized in Table 2.

**Table 1:** Maize genes encoding SUN-domain proteins.

	Gene			mRNA	
	
Class	Maize gene^a^	Locus^b^	BAC^c^	cDNA^d^	UniGene^e^
CCSD	ZmSUN1	5 S, bin 5.04	AC217313	EU964563	Zm.94705
	ZmSUN2	3 S, bin 3.04	AC197221	BT055722	Zm.6043
PM3	ZmSUN3	3L, bin 3.06	AC195254	GRMZM2G122914_T01	
	ZmSUN4	8L, bin 8.06	AC188196	GU453173	Zm.17612
	ZmSUN5	8L, bin 8.05	AC194341	EU953247	Zm.31400

^a^Gene names assigned in this manuscript. Numerical designations (ZmSUN1-5) do not

necessarily imply orthology with similarly named genes in other species.

^b^Chromosome number and arm (S, short; L, long), genetic bin as designated for the UMC 1998 linkage map [56].

^c^GenBank accession numbers for B37 BACs that include the indicated SUN gene.

^d^Best corresponding full-length cDNA or gene model from B73 RefGen_v1; ZmSUN4 is from maize line W23, all others from B73.

^e^GenBank maize UniGene accession numbers.

**Table 2:** Properties and motifs of maize SUN-domain protiens.

	Predicted properties^a^					Motifs^e^			
	
Class	Name	Length^b^	kDa	pI^c^	Cys^d^	TM^f^	SUN^g^	CC^h^	PAD^i^
CCSD	ZmSUN1	462	51	9.1	3	W116-W141	N315-K454, (6 e-39)	F165-D228	
	ZmSUN2	439	48	7.8	3	T84-W109	P294-G425 (3 e-32	D166-L192	
PM3	ZmSUN3	613	68	4.9	7	TM1, L33-V55 TM2, L555-M577 TM3, L599-I612	F233-D357 (2 e-38)	A482-F515	G437-G474
	ZmSUN4	639	71	5.2	9	TM1, G58-L75 TM2, L581-M603 TM3, G621-I638	F257-D381 (7 e-38)	D514-E539	G463-G500
	ZmSUN5	589	64	5.3	9	TM1, V46-L66 TM2, L525-C544 TM3, M572-Y588	H197-D321 (9 e-35)	CC1, V414-E434 CC2, K495-K523	G407-G444

^a^Protein ORFs used were predicted from the sequences listed under cDNA from Table 1. Properties were calculated by means of the online ProtParam software, http://us.expasy.org/tools/protparam.html[80].

^b^Total number of amino acids in the predicted ORF.

^c^pI, predicted isoelectric point.

^d^Total number of cysteine residues.

^e^Motifs and domains are indicated by the first and last amino acid; the amino acid numbers for the ORFs are those from the sequences listed under cDNA from Table 1.

^f^TM, locations of transmembrane regions predicted by the online software www.ch.embnet.org/software/TMPRED_form.html[70]. The multiple TMs of the PM3 proteins are named TM1, TM2, and TM3 according to the order of their occurrence starting from the N-terminus.

^g^SUN, Sad1_UNC superfamily (pfam07738) domain locations and significance values are from alignments to the Conserved Domain Database (CDD version 2.21, NCBI), http://www.ncbi.nlm.nih.gov/Structure/cdd/cdd.shtml[81].

^h^CC, coiled-coil motifs are predicted from the online COILS software http://www.ch.embnet.org/software/COILS_form.html[69]. The two CCs in SUN5 are called CC1 and CC2 according to the order of their occurrence starting from the N-terminus.

^i^PAD, PM3-associated domain of unknown function defined here by multiple sequence alignments.

### Conservation of Two Classes of SUN-domain Proteins in Plants

**Figure 1:**
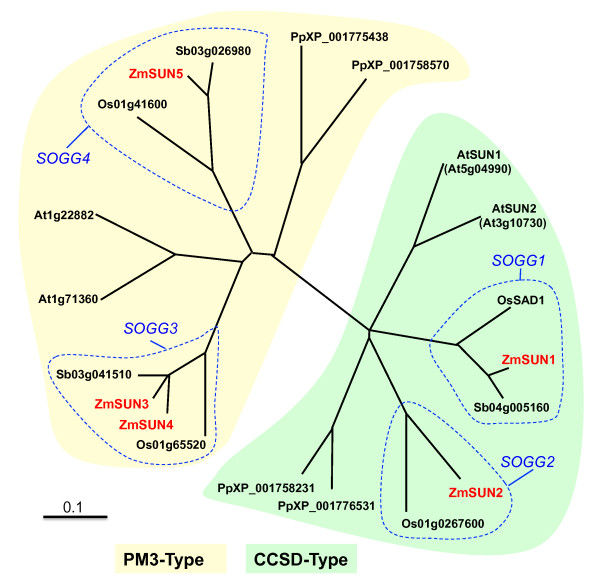
**Phylogenetic relationships among selected SUN-Domain proteins in the plant kingdom**. An unrooted phylogenetic tree of SUN-domain proteins is shown, deduced from full-length cDNAs from maize (*Zea mays*, Zm), *Arabidopsis *(At), rice (Os), *Sorghum bicolor *(Sb), and moss (*Physcomitrella patens*, Pp). GenBank accession numbers are given in the figure, except for those of maize, which are from sequences listed in Table 1. The protein maximum-likelihood tree was created with TreeView, version 1.6.6 [71]. Proteins belonging to the canonical (CCSD, green shaded area) and mid-SUN (PM3, yellow shaded area) classes are indicated. Four SUN orthologous grass groups (SOGG1-4) are also indicated. A partial EST from sorghum (Sb03g010590/PUT-157a-Sorghum_bicolor-11155) aligns with the SOGG2 group but was excluded from the analysis because it lacked a full-length ORF. Scale bar (0.1) represents 10 expected amino-acid changes for every 100 residues.

We next carried out a phylogenetic analysis of CCSD and PM3-type SUN-domain protein sequences from maize, sorghum, rice, *Arabidopsis*, and moss (*Physcomitrella patens*). Protein sequence alignments were used to produce an unrooted phylogenetic tree, shown in Figure 1. From the unrooted phylogenetic tree, we observed two different types of groupings. The first, a clear separation of the CCSD (green shaded area, Figure 1) and PM3 (yellow shaded area, Figure 1) subfamilies, suggests an ancient divergence of these two classes. These data also suggest that the PM3 proteins originated early in the life of the plant kingdom, predating the origin of flowering plants. The second, four orthologous groups observed within the grass species (SUN Orthologous Grass Groups, labeled SOGG1-SOGG4 in Figure 1), may reflect functional divergence within each subfamily. If so, these SOGGs would be predicted to share expression patterns or genetic functions. Interestingly, the two plants outside the grass family, *Arabidopsis *and the nonflowering tracheophyte *P. patens*, also have genes predicted to encode at least two CCSD and at least two PM3 proteins, but their relationship to the SOGGs is not resolved by this phylogenetic analysis. Plant genomes therefore appear to encode two different multigene subfamilies of SUN-domain proteins, the CCSD and PM3 types.

### Shared Gene Structures Reflect an Early Divergence of the Two Types of Maize SUN-domain Proteins

The 2.3-Gb maize genome is partitioned among 10 structurally diverse chromosomes, which are predicted to encode over 32,000 genes [53]. The genetic map of maize is subdivided into approximately 100 10-to 15-cM bins [56]. The genome is complex and dynamic because of extensive and recent large segmental duplications [53,57-59] and a major expansion of long terminal repeat sequences over the last few million years. Current breeding lines and natural accessions of maize harbor large amounts of sequence diversity and many structural polymorphisms [53,58,60].

**Figure 2:**
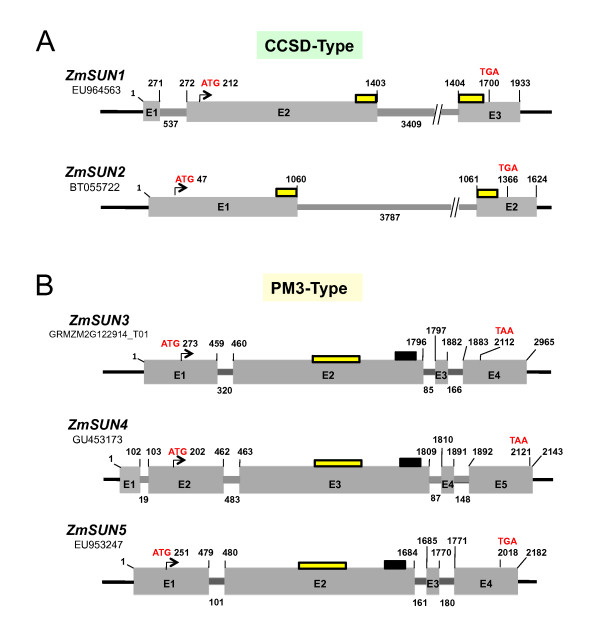
**Genomic structures for the two subfamilies of maize SUN-domain protein genes**. The locations of exons, start (ATG), and stop (TGA, TAA) codons are shown for each gene. The diagrams were drawn from predictions made by the SPIDEY program http://www.ncbi.nlm.nih.gov/spidey/ on the basis of alignments of cDNA to genomic DNA sequences (from Table 1). The mRNA coordinates for the exon bases are listed above the diagrams. Exons are numbered, and the intron lengths (bp) appear below the diagrams. (A) The canonical C-terminal SUN domain genes show a large intron at a conserved location interrupting the SUN domain region (yellow box) within the ORF. (B) The plant-prevalent mid-SUN 3 transmembrane genes all share a large exon that contains the entire SUN domain plus a domain of unknown function (black box) associated with these genes, as well as two small introns before the last exon.

Using full-length cDNAs (listed in Table 1) together with the B73 reference genome, we were able to define the structures of five maize SUN-domain genes as shown in Table 1 and Figure 2. Three of these genes *(ZmSUN1, 2, and 3*) are distributed as unlinked loci that map to two different chromosomes; *ZmSUN4 *and *ZmSUN5 *reside in adjacent genetic bins. In determining whether the CCSD or PM3 genes were located in any of the known blocks of genome duplication, we found that the high degree of sequence similarity between the SOGG3 genes *ZmSUN3 *and *ZmSUN4 *suggests they arose as part of a gene-duplication event that is known to have resulted in many closely related gene pairs in maize [56,58]. Indeed these two genes reside within a large syntenic duplicated block on chromosomes 3 (bin 3.06) and 8 (bin 8.06). This observation is consistent with the phylogenetic results that revealed the presence of four orthologous SUN-domain protein groups, SOGG1 (*ZmSUN1*), SOGG2 (*ZmSUN2*), SOGG3 (*ZmSUN3*, *ZmSUN4*), and SOGG4 (*ZmSUN5*). Surprisingly, we have not observed duplicate genes for *ZmSUN1*, *ZmSUN2*, or *ZmSUN5*, so these may exist as single copies in the B73 maize genome.

An analysis of intron and exon structures within the maize *SUN *genes showed that the gene structures are conserved within each class. The CCSD genes had two or three exons, and the SUN domain was split between the exons. On the other hand, the PM3 genes had 4-5 exons and a SUN domain that was encoded within the largest exon. Comparative analysis of the maize *ZmSUN *gene structures revealed that the CCSD genes shared an ancestral intron that interrupts the SUN domain (between K364 and V365 in the ORF of *ZmSUN1 *and between K338 and D339 in the ORF of *ZmSUN2; *Figure 2A). This ancestral intron position may be a hallmark of this class of *SUN *genes, as it is also found in the *Arabidopsis*, rice, sorghum, and moss homologs. *ZmSUN1 *and *ZmSUN2 *share a large intron, greater than 3 kb in size, whereas the PM3 genes all possess small introns ranging from 19 to 483 nucleotides in size.

### Properties of Maize SUN-domain Proteins

**Figure 3:**
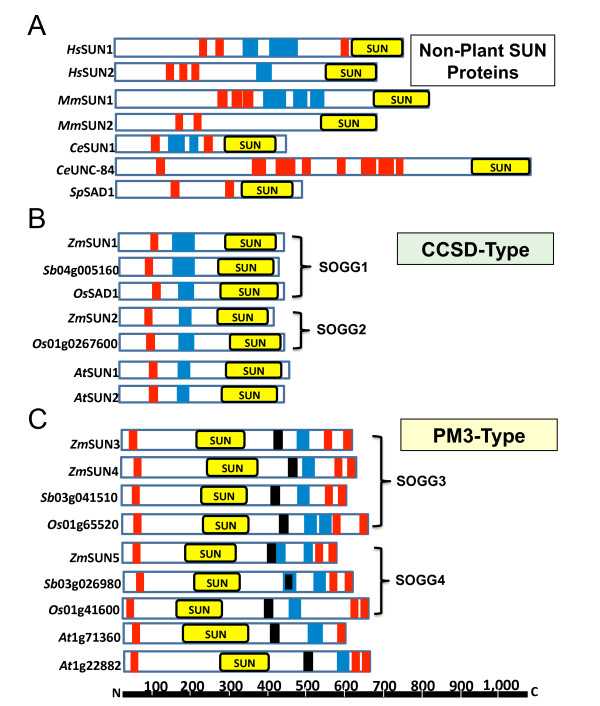
**Conservation of functional domains in plant and animal SUN-domain proteins**. Comparative diagrams of SUN-domain proteins depicting protein sizes and domain locations (see Table 2). The positions of transmembrane (red), coiled-coil (blue), SUN (yellow), and PM3-associated (PAD) domains (black) are indicated for each protein. (A) Known nonplant SUN-domain proteins (human, Hs; mouse, Mm; nematode; Ce; fission yeast, Sp) of various sizes, but all with a single C-terminal SUN domain are shown (UniProt accession numbers: HsSUN1, O94901; HsSUN2, Q9UH99; MmSUN1, Q9D666; MmSUN2, Q8BJS4; CeSUN1, Q20924; CeUNC84, Q20745; SpSAD1, Q09825). (B) CCSD and (C) PM3 plant proteins grouped by their orthologous groups (see Figure 1).

Using the full-length cDNAs listed in Table 1 we predicted the encoded proteins for five different maize SUN-domain proteins. Their features and primary motifs are summarized in Table 2 and diagrammed in Figure 3. A multiple sequence alignment of CCSD-type proteins reveals divergence at the N-terminal region and conservation at the C-terminal region which encompasses the SUN domain (Additional file 1 Figure S1). Several previously characterized fungal and animal SUN-domain protein structures (Figure 3A) are also shown for comparison. The SUN-domain proteins of human, mouse, worm, and fission yeast differ in size and number of transmembrane and coiled-coil motifs, but all a have single C-terminal SUN domain, considered a diagnostic feature for this family of NE-associated proteins. The plant proteins that most closely resemble the founding members of the SUN-domain protein family are those encoded by the CCSD genes. The plant CCSD proteins exhibit conserved size and overall structure to a remarkable degree, having one transmembrane domain followed by one coiled-coil domain, and share an overall size of about 50 kDa (Figure 3B). Relatively little is known about the CCSD proteins in plants. Fluorescent protein fusion assays with AtSUN1, AtSUN2, and OsSad1 demonstrate localization to the NE [18,55]. In addition, The CCSD proteins probably share some functions with their animal counterparts but have not been proven to do so.

Even less is known about the PM3 proteins, and their functions are completely uncharacterized. They are significantly larger than plant CCSD proteins (Figure 3C). Their shared structural features are an N-terminal transmembrane domain, an internal SUN domain, a PAD, one or more predicted coiled-coil motifs, and two closely spaced C-terminal transmembrane domains (Table 2 Figure 3C). This collection of features defines them structurally, but the central location of the SUN domain is not unique to plants. Other, nonplant mid-SUN-domain proteins, largely uncharacterized, from various species including fungi, flies, worms, and mammals can be identified by sequence-search analyses (data not shown). Whether or not these proteins reside or function in the NE remains to be determined.

**Figure 4:**
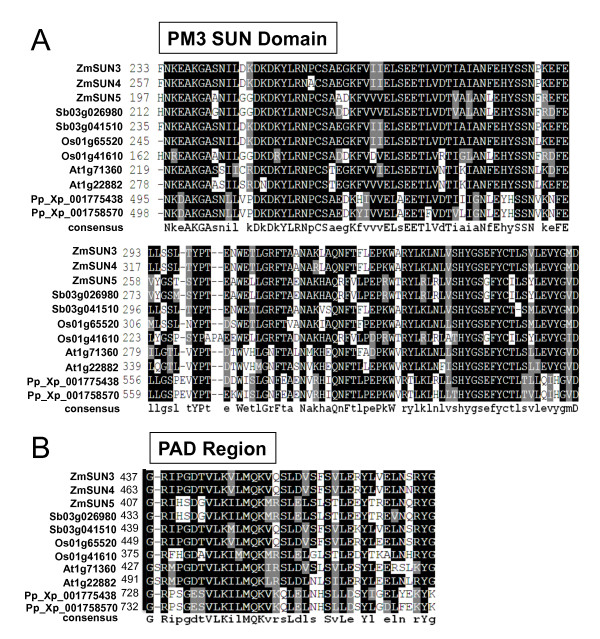
**Multiple sequence alignment of PM3 SUN domains and PAD regions**. Multiple sequence alignments from ClustalW2 for isolated domains of PM3 proteins from five plant species. Box shade alignment displays show conserved residues (identical black, similar grey) and an alignment consensus sequence at the bottom. (A) Alignment of the SUN domains with amino-acid numbers indicated. (B) Alignment of PAD regions composed of a ~38-amino acid segment.

In addition to their difference in size and SUN domain locations, these protein subfamilies are distinct in other interesting ways (Table 2). The CCSD-type proteins have a basic isoelectric point, whereas the PM3-type proteins have an acidic one (Table 2). In addition, the PM3 proteins have a relatively large number of cysteine residues that may play important roles in intra- or intermolecular disulfide bridge formation. Furthermore, a multiple sequence alignment reveals that the PM3 proteins all have the highly conserved region that we call the PAD (Figure 4 Additional file 2 figure S2). This region of approximately 38 residues appears diagnostic for plant PM3 proteins and is spaced about 80-90 residues after the SUN domain. The SUN domain and the PAD for 11 plant proteins revealed a high degree of amino acid conservation.

Despite the similarity of domain architecture and sequence similarity within conserved domains, the remainder of the protein regions exhibit considerable sequence divergence between the SOGG3 and SOGG4 members in any given species. Overall, these analyses show that the maize genome encodes at least two multigene families of SUN-domain proteins. Each of these two subfamilies comprises at least two genes. *ZmSUN1 *and *ZmSUN2 *are CCSD-type and are most closely related to plant SUN-domain homologs *AtSUN1*, *AtSUN2*, and *OsSad1*. *ZmSUN3, 4*, and *5 *are PM3-type and probably represent a previously unknown class of SUN-related proteins in plants.

### mRNA Expression Profiling of *ZmSUN *Protein Genes

The conservation of the SUN-domain protein genes in plants suggests that they potentially have functions similar to those of their animal counterparts, for example nuclear positioning and motility within the cell, bridging the cytoplasm to the cortical layer of the nucleoplasm, and contributing to meiotic chromosome segregation through telomere tethering before synapsis and recombination [8,9,44]. Maize *SUN *domain genes that function in basic somatic cell processes such as mitosis, nuclear architecture, and chromosome tethering might be expected to show ubiquitous expression, whereas those that function in meiosis or pollen-nuclear migration or nuclear fusion at fertilization might show a more limited expression profile, being active in reproductive organs such as flowers, egg and pollen mother cells, and gametophytic tissues such as pollen grains. To begin to examine these possibilities, we looked at gene expression at the mRNA abundance level using three different sources of information: NCBI's UniGene; microarray expression data from anthers, which contain male meiotic cells; and Solexa transcriptome profiling data derived from maize inbred line B73 tissues.

Four of the five genes (all but *ZmSUN3*) are represented by consensus UniGene models in NCBI (Table 1), and three of these, *ZmSUN1*, *ZmSUN2*, and *ZmSUN4*, are accompanied by quantitative EST profile information expressed as transcripts per million, which we converted to transcripts per ten million (TPdM). The EST data were pooled according to tissue type, and only relatively deeply sequenced libraries (10,000-15,000 or more) showed evidence of expression, as summarized in Additional file 3 Figure S3. The CCSD genes, *ZmSUN1 *and *ZmSUN2*, appeared to be expressed at relatively low levels (200-2,000 TPdM) in several tissues, including ear, endosperm, embryo, meristem, pollen, and tassel. Only one PM3-type SUN-domain gene, *ZmSUN4*, currently has corresponding EST profile data available from NCBI. It too shows relatively low expression levels (~400-3,000 TPdM) in a variety of tissues, such as embryo, pericarp, and shoot. These values are roughly 10% of those for UniGene EST data from two control so-called house-keeping genes, alpha tubulin 4 (*tua4*, Zm.87258) and cytoplasmic GAPDH (Zm.3765), which are expressed in 17 of the 19 tissues at levels from ~2,200 to 21,000 TPdM.

**Figure 5:**
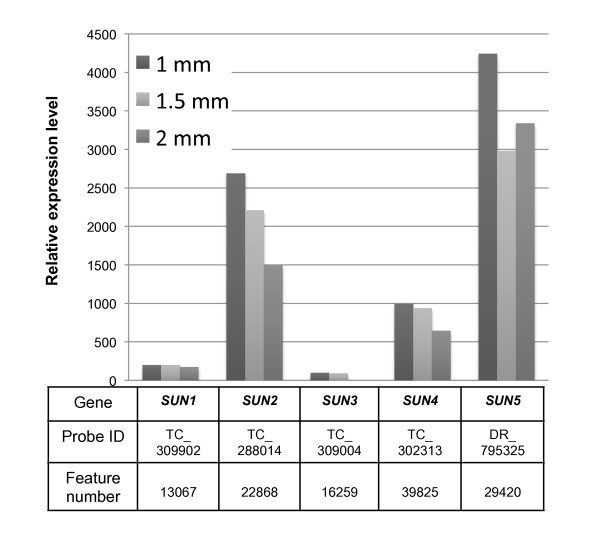
**Expression of *ZmSUN *genes in meiosis-stage anthers**. Relative expression levels shown by maize SUN-domain protein genes obtained from published microarray experiments (Gene Expression Omnibus [73,79]). The cDNAs were from meiosis-stage anthers 1 mm, 1.5 mm, and 2 mm in length. The histogram depicts signals relative to the whole-chip mean. Dye-normalized values for each channel generated by Feature Extraction software were divided by the median intensity for that channel on each array, and then the log base 2 was taken, as previously described [61]. The table at the bottom tabulates the gene name (Gene), Probe ID (the gene model/contig being targeted), and feature number (chip oligo 60-mer).

Given the role of SUN-domain proteins in meitoic telomere behavior in a variety of nonplant eukaryotic species, we next examined microarray data from mRNA expression profiles of male reproductive organs from 1- to 2-mm anthers. Anthers in this size range are from tassels that had not yet emerged and and contain meiocytes before or during meiotic prophase. Microarray probes (60-mer oligonucleotides, as described in [61]) that showed 100% match with our B73 gene models were available for each gene, and their relative expression values are plotted in Figure 5. From these analyses, we observed that the relative expression levels of *ZmSUN5 *and *ZmSUN2 *were highest in meiosis-stage anthers, whereas *ZmSUN1 *and *ZmSUN3 *were the lowest there, and *ZmSUN4 *was intermediate in the overall range (~80 to 3,000 TPdM).

Ascribing the meiotic telomere clustering functions to any one of the five *SUN *genes may prove difficult, at least partly because the anther is made up of several different cell types that include not only cells in meiosis but also a layer of epidermal, intermediate, and tapetal cells. The expression or function of plant *SUN *genes could be partitioned among these cell types, whereas these methods produced only a single value over the entire anther [61]. Another consideration is that even single cells may contain multiple SUN proteins with different, related, or even cooperative functions, such as NE rearrangements, interaction with nuclear pores, or paternal storage of gene products for postmeiotic functions such as pollen mitosis, pollen tube growth, nuclear migration, and fertilization.

### Solexa Transcriptome Expression Profiling

**Figure 6:**
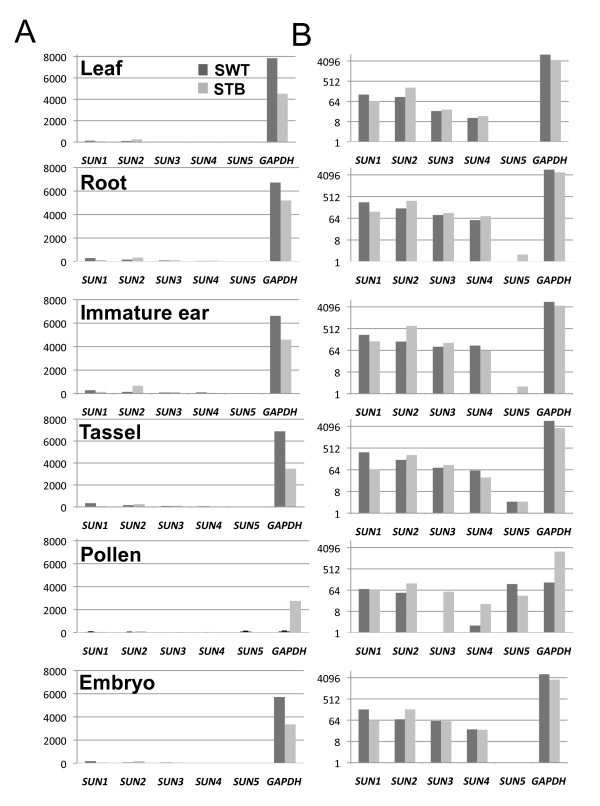
**Expression profiling of *ZmSUN *genes by Solexa tag-based and whole-transcriptome sequencing**. mRNA from various B73 tissues was subjected to two Solexa sequencing platforms, Solexa whole-transcriptome (SWT) and Solexa dual-tag based (STB). The vertical axis represents the number of 36-nt (SWT) or 21-nt (STB) sequence tag matches per ten million transcripts. (A) Expression levels of *ZmSUN *genes and the control gene, cytoplasmic *GAPDH*, are graphed for comparison. (B) The same data are plotted as semi-log_2 _for easier comparisons among the low-expression *ZmSUN *genes.

Expression levels for the two Solexa-based sequencing-by-synthesis methods we used, Solexa dual-tag-based (STB) and Solexa whole transcriptome (SWT) http://www.illumina.com/technology/sequencing_technology.ilmn), are also reported in transcripts per 10 million and derived from experiments on pooled samples of six major tissues of the B73 cultivar. Both the Solexa technology and the EST UniGene data provide discrete counts of sequenced molecules, but the Solexa data are based on millions, not thousands, of reads per experiment, providing better representation of genes such as the *ZmSUN *genes that were expressed at low levels in each organ. The two platforms gave similar results for pooled tissue samples, as summarized in Figure 6 and tabulated in Additional file 4 Table S1. Most of the SUN genes were expressed at low levels across multiple tissues; expression was similar within tissue types, regardless of developmental stage. The *ZmSUN *gene expression levels were about 2% of those of the moderately expressed housekeeping control gene, cytoplasmic glyceraldehyde 3-phosphate dehydrogenase (*GAPDH*, Figure 6).

To show more clearly the variation in expression levels among the *SUN *genes, we replotted the same data as semi-log_2 _(Figure 6B). The overall expression pattern is consistent with basic functions for SUN-domain proteins in most cell types. A notable exception to the widespread pattern of expression was that of *ZmSUN5*, which showed a very distinct and much more restricted pollen-related pattern of expression (Figure 6 pollen). Such an expression profile predicts that *ZmSUN5 *should be required for specialized processes such as nuclear migration down the pollen tube and possibly double fertilization. An interesting and related observation is that fertilization involves nuclear fusion, as does karyogamy, which in yeast involves active nuclear migration and SUN-domain proteins [9,38,62].

The present report represents the first description of relative mRNA expression levels of all members of a *SUN *gene family in any plant species and may therefore prove useful to investigators of the functions of plant SUN-domain proteins. Despite some variation in the data across different expression platforms, as summarized above, a consistent trend for most of the *ZmSUN *genes is that they are expressed in many different tissues at relatively low levels, a finding similar to that of Graumann *et al*. [19] for the CCSD-type *AtSUN2 *gene. In addition, we observed a distinct exception to this overall pattern with *ZmSUN5*, whose expression appears to be highly specific to pollen. Given the lack of information on PM3-type SUN proteins, we set out to characterize this group further in plants. We chose to examine a PM3-type gene that was expressed in many cell types including those expressed in meiosis-stage anthers with possible roles in meiotic telomere functions.

### Isolation and Characterization of a Maize PM3-type SUN-Domain Protein Gene from a Meiotic cDNA Library

The role of *SUN *genes in telomere-associated recombination and crossover control has been established for animals and yeast and is likely to exist in plants as well [33,63,64]. In this regard, we find intriguing that two different laboratories [65,66] recently and independently mapped a recombination control QTL in maize to bin 3.06, where *ZmSUN3 *resides. We screened a meiosis-enriched cDNA library for *ZmSUN3 *and its closely related duplicate *ZmSUN4 *using a 639-bp PCR product corresponding to a region of the SUN domain of *ZmSUN3 *at a stringency of Tm-15°C. The probe has a high degree of similarity to both *ZmSUN3 *and *ZmSUN4 *yet it is not similar enough to *ZmSUN5 *or either of the CCSD-type genes to detect them. From approximately 500,000 plaques, we isolated two identical full-length cDNA clones of *ZmSUN4 *with identical insert sequences. The detection of *ZmSUN4 *but not *ZmSUN3 *is consistent with the relative expression levels for *ZmSUN3 *and *ZmSUN4 *in meiosis-stage anthers (Figure 5).

**Figure 7:**
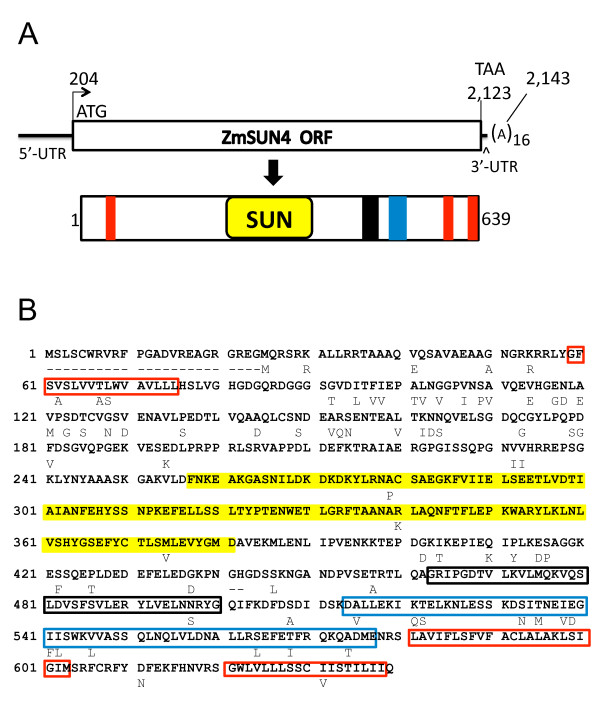
***ZmSUN4 *cDNA and protein features**. (A) *ZmSUN4 *(genotype W23) full-length cDNA, showing the 5' and 3' UTRs, open reading frame (ORF), and poly-A tail. A diagram of the protein indicates domain locations as described in Figure 3. (B) Annotated protein sequence predicted from full-length cDNA ORF (GenBank GU453173). Color scheme is the same as in Figure 3. Amino acid residues below the ZmSUN4 sequence show divergent residues of the duplicated locus on chromosome 3L, *ZmSUN3*, genotype B73.

The full-length cDNA sequence for *ZmSUN4 *[GenBank: GU453173] and the deduced protein sequence and motifs are illustrated in Figure 7A. The predicted protein sequence from the ZmSUN3 gene is also shown (Figure 7B) and reveals that the B73 SUN3 and W23 SUN4 are 88% identical. This relatively high level of protein similarity reflects their divergence after a maize genome duplication event estimated to have occurred about 5-12 mya [53]. The extent which these proteins have evolved functionally remains unknown.

The W23 *ZmSUN4 *full-length cDNA is 2,158 bp in length and has a predicted open reading frame (ORF) of 1,920 bp encoding a 639-residue protein with a predicted molecular mass of ~71 kD and an acidic isoelectric point of 5.2 This full-length *ZmSUN4 *cDNA predicts a protein with all of the motifs and arrangents (Table 2 Figure 7B) that are typical of the entire class of PM3 proteins.

### Localization of a Maize PM3-type Protein

**Figure 8:**
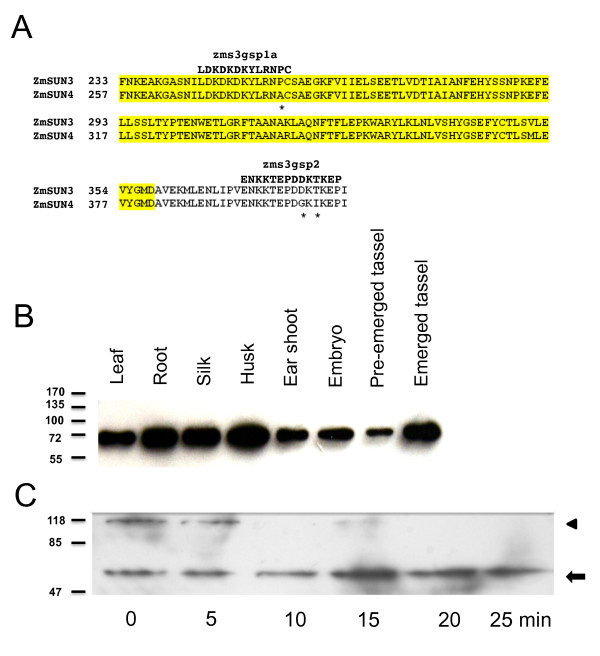
**Western blot of proteins ZmSUN3 and ZmSUN4**. (A) Two peptide antibodies were made against synthetic peptides within (zms3gsp1a) and just after (zms3gsp2) the SUN-domain of the maize ZmSUN3 protein. The corresponding regions in ZmSUN3 and ZmSUN4 are aligned, and asterisks indicate divergent residues in ZmSUN4. (B) Western-blot detection (top panel) of ZmSUN3 and ZmSUN4 in various plant tissues. Protein was loaded on an equal-fresh-weight basis for leaf, root, silk, husk, earshoot, embryo, meiosis-stage tassel, and postmeiotic tassel, resulting in the detection of a single band of ~72 kDa. (C) Immunoblot showing the effect of increased sample boiling time on bands detected. Protein from meiosis-stage anthers appeared as a single band at ~70 kDa (arrow) after the protein was boiled in SDS for 10 min or more.

**Figure 9:**
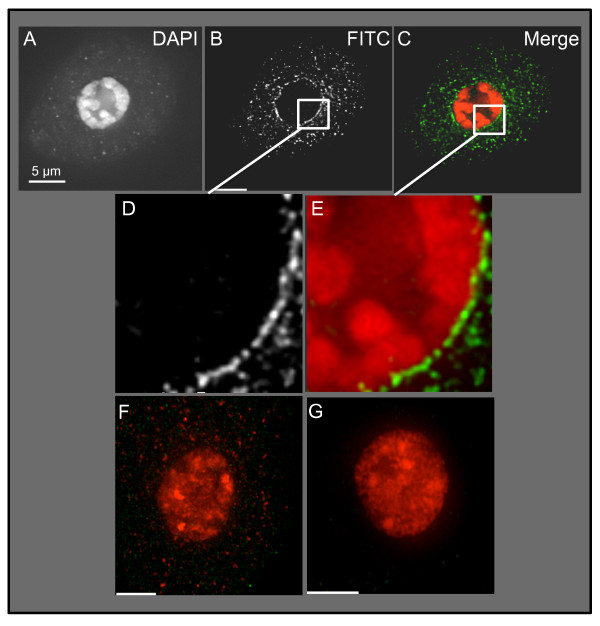
**Immunolocalization of PM3 SOGG3 Proteins at the nuclear periphery**. Combined antisera (zms3gsp1a and zms3gsp2) or preimmune control sera were used to stain formaldehyde-fixed uninucleate pollen mother cells. The immune complex was visualized by deconvolution microscopy in the FITC channel with A488-goat-anti-rabbit sera. Images from a single cell are shown. (A-C) Projection of the central 2/3 of the three-dimensional set of data shows DAPI image (A), FITC image (B), and pseudocolor overlay (C). Zoom up of a region of the nucleus-cytoplasm boundary is shown for the FITC (D) and overlay (E) images. Control staining with preimmune sera (F) or secondary only (G) are shown with a color scheme (red DAPI, green FITC) and scaling parameters that match those of panel C.

To test for the presence and localization of ZmSUN3/4 proteins *in planta*, we developed peptide antibodies for western blotting and immunolocalization, and the results are summarized in Figure 8 and 9. The peptides used and the corresponding ZmSUN3/4 sequences are shown Figure 8A. Our survey of a variety of tissues for the presence of PM3-type proteins with antisera to zms3gsp1A (Figure 8B) revealed only one band band of about 70 kDa in all of the tissues surveyed, including leaf, root, silk, husk, earshoot, embryo, preemergence (meiotic) tassels, and emerged (postmeiotic) tassels. This broad detection is consistent with the mRNA expression profiles for *ZmSUN3 *and *ZmSUN4 *(Figure 5 and 6).

Our examination of proteins from isolated male flowers at meiotic stages of development detected high-molecular-weight bands that were considerably larger than the predicted protein sizes. Given the number of cysteine residues and the possibility of disulfide bridges, we examined the effect of prolonged boiling times in the presence of reducing agents (0.1 M 2*-*mercaptoethanol, 10% SDS) on the detectable band patterns. These high-molecular-weight bands were not detected in the protein samples examined for multiple other, different, nonanther tissues (Figure 8B). The basis for this difference is not known, but it may result from more highly cross-linked SUN3/4 protein in the extracts from anthers than in those from the other tissues. After 10 or more minutes of boiling, the antibodies detected a single band of about 70 kDa (Figure 8C), similar to those detected in the multitissue survey blot (Figure 8B). Therefore, ZmSUN3, ZmSUN4, or both appear to be present in meiosis-stage anthers.

Our examination of formaldehyde-fixed cells, shown in Figure 9 revealed the strongest staining around the nuclear periphery but also detected considerable speckled cytoplasmic staining in a postmeiotic uninucleate pollen mother cell. The cytoplasmic staining may reflect nonspecific background or true signal from ER-localized PM3-type SUN-domain protein. Interestingly, we have yet to detect staining in meiotic prophase nuclei with these antibodies, possibly because of difficulty in the preservation conditions or in detecting the epitope in prophase nuclei or possibly because of an absence of PM3-type SUN-domain proteins in meiotic cells. The results of negative control experiments, using preimmune sera and secondary antibody only, are shown in Figure 9 at image scaling comparable to that used for the anti-PM3-antibody staining (Figure 9C). The lack of staining in the controls suggests that the staining patterns noted with the anti-PM3 sera were specific.

These data provide the first direct evidence of a PM3 SUN-domain protein localized to the nuclear periphery and suggest that this SUN domain in this subfamily of plant proteins can reside in the NE like the CCSD proteins. Together, these observations suggest that plant nuclei contain multiple different SUN-domain proteins.

### Models of the Topology of Plant SUN-domain Proteins

**Figure 10:**
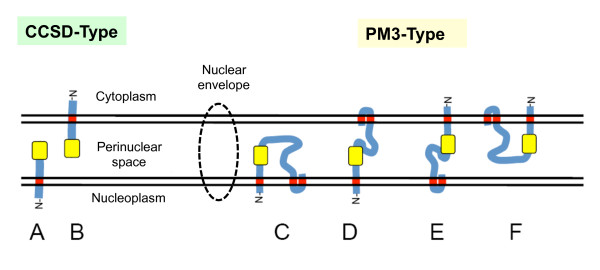
**Maize SUN topology models relative to the membranes of the nuclear envelope**. Possible protein arrangement models with the SUN (yellow) domain in the perinuclear space are shown for the CCSD (A-B) and PM3 (C-F) proteins. Models do not attempt to depict multimer interactions that may occur with the SUN or coiled-coil (not shown) domains.

The two structural classes of plant SUN-domain proteins found in maize, and shown to be occur commonly in many plant species, may have different functions. If they serve as physical connectors that transduce forces from the cytoplasm to the nucleus, determining their topologies and dispositions relative to the membranes of the NE will be an important step toward elucidating their biological roles. Several models of different topoligical arrangements for generalized CCSD and PM3 SUN proteins in the plant NE are presented in Figure 10.

If CCSD SUN proteins adopt a configuration like that of plant, animal, or fungal SUN proteins, the most likely arrangement would be that depicted by topology model "A" in Figure 10. In this configuration, the N-terminus would be in the nucleoplasm, possibly interacting with chromatin, inner-nuclear-membrane-associated proteins, or telomeres, and the SUN domain would be positioned within the perinuclear space. Connections to the cytoplasm would require interactions with other proteins embedded in the outer nuclear membrane. The configuration depicted in topology model "B" would suggest an opposite set of interactions. Given the structure of the NE, the two models are not necessarily exclusive, as the two membranes are continuous and fused around nuclear pore complexes.

For the PM3 SUN proteins, four different models (Figure 10) are presented for consideration because three transmembrane domains are involved. The C-terminal transmembrane domains are close together and unlikely, although not necessarily unable, to traverse the entire lumenal space. Only models with the last two transmembrane domains in the same membrane are therefore presented. Of these, topology models "D" and "E" are intriguing in that they predict a single protein bridge with both nucleoplasmic and cytoplasmic segments. Topology model "C" could have two different nucleoplasmic segments and thereby serve as a scafold for multiple nuclear molecules or complexes, including chromatin and nonchromatin nuclear proteins, other NE proteins, or telomeric DNA. Similarly, topology model "F" depicts a protein with two cytoplasmic segments that might be capable of interacting with two cytoplasmic partners, while requiring additional protein interaction to form a functional nucleoplasmic-cytoplasmic bridge.

In nonplant systems, SUN proteins are linked to the cytoplasm by an interaction with KASH-domain proteins that traverse the outer nuclear membrane. The KASH domain proteins connect to various cytoskeletal components to function as cargo-specific cytoskeletal adaptor proteins [13,42,67]. As a family, the KASH domain proteins have limited homology over a small portion of their entire protein sequence, and no plant KASH-domain protein homologs have been identified by sequence analyses thus far. Genetic or protein interaction screens may be required to identify SUN-interacting partners and their function in plants.

## Conclusions

The maize genome encodes a family of SUN-domain protein genes that form two distinct classes; the CCSD-type, resembling canonical SUN-domain proteins, and the PM3-type, representing a novel structural class shown here to be expressed in multiple tissues of maize and concentrated at the nuclear periphery in pollen mother cells. These two subfamilies are found in flowering plants and moss and therefore probably originated early in plant evolution, if not before that. The discovery of this gene family opens new avenues for investigation of molecular mechanisms that may link nuclear architecture to chromatin dynamics and nuclear positioning in maize. Future genetic analyses will be important for defining the biological role of these plant *SUN *genes *in vivo*.

## Methods

### Bioinformatics and *SUN *Gene Models

The B73 reference maize genome http://www.maizesequence.org was queried with SUN-domain protein sequences from *C. elegans *Unc-84 [GenBank: NP_001024707], *S. pombe *Sad1p [GenBank: NP_595947], and rice Sad1 [GenBank: NP_001055057], which identified CCSD protein genes (*ZmSUN1 and ZmSUN2*). Further BLAST searches with these sequences led to the identification of PM3 genes (*ZmSUN3, 4*, 5). Genomic DNA structures for *ZmSUN *genes were produced with full-length cDNAs, ESTs, or EST contigs with B73 genomic DNA with SPIDEY, http://www.ncbi.nlm.nih.gov/spidey. The genomic structure for *ZmSUN3 *was determined from available EST assembly data at PlantGDB http://www.plantgdb.org/, as no full-length B73 cDNA clone was available at the time. Protein parameters including amino acid length, molecular weight, and isoelectric points were obtained from ExPASy [68]. Secondary structure domains, including the locations of the SUN domain, predicted coiled coils, and predicted transmembrane regions, were obtained from the NCBI conserved-domain database (version 2.21), COILS [69], and TMpred [70] prediction software respectively. The PAD located in *ZmSUN3, 4*, and *5 *was identified by analysis of a multiple sequence alignment of full-length proteins of maize, *Arabidopsis*, sorghum, rice, and a moss (*P. patens*) with ClustalW2 http://www.ebi.ac.uk/Tools/clustalw2/. The phylogenetic tree displayed in Figure 1 was created by ClustalW2, with the default multiple-sequence-alignment matrix (Gonnet 250) and is displayed as an unrooted maximum-likelihood tree from TreeView, version 1.6.6 [71].

### mRNA Expression Analyses of *ZmSUN *Genes

Expression data for mRNA levels was extrapolated from three different sources. For the UniGene EST, expression profiles are computed relative abundance values derived from NCBI's UniGene for *ZmSUN1 *(Zm.94705), *ZmSUN2 *(Zm.6043), and *ZmSUN4 *(Zm.17612). For the anther microarray data, relative expression levels were extracted from microarray experiments available at NCBI (Gene Expression Omnibus, http://www.ncbi.nlm.nih.gov/geo/[72,73]). The cDNAs were originally obtained from meiosis-stage anthers that were 1 mm, 1.5 mm, or 2 mm in length. Probe signals for *ZmSUN *genes were determined as previously reported [61]. For transcriptome analysis, Poly(A+) RNA was isolated from various maize tissues with Trizol (Gibco, BRL), Qiagen, MACS (Miltenyi Biotec), and FastTrack (Invitrogen) RNA isolation kits. Two Solexa-based transcript-quantification platforms were used to measure the abundance of *SUN *transcripts, the Solexa shole-transcriptome and Solexa dual tag-based methods [74,75]. Both of these technologies involve 36-nt or 21-nt sequence read lengths produced from multiple locations in the transcripts. The whole-transcriptome data were not restriction-enzyme anchored, so the multiple 36-nt sequences were spread along the transcripts. For the dual-tag-based methods two four-base cutter restriction enzymes, *Dpn*II and *Nla*III, were used as initiation sites for the 21-nt sequences, and therefore deep transcript counts were obtained from fewer sites in the transcripts. Only repetitive sequence reads found at 10 or fewer distinct locations in the B73 genomic sequence (by comparison to 17,455 publicly available B73 BAC sequences) were used in determining the relative gene expression levels. Sequences found more than 10 times in the genome were classified as repetitive sequences and were excluded from the analysis. The *GAPDH *cytoplasmic gene is known to have a moderate and relatively ubiquitous expression level in many maize tissues [76] and is included for comparison. The dual tag-based analysis was carried out with an Illumina GA2 machine and cDNAs treated with two restriction enzymes, *Dpn*II and *Nla*III. The aggregate counts of the resulting sequence reads from these sites, excluding repetitive sequences, were used to quantify the overall gene expression level, reported here in parts per ten million transcripts.

### Molecular Cloning and Sequence Analysis of a Maize Full-Length SUN cDNA, *ZmSUN4*

A full length maize PM3-type SUN cDNA was isolated by hybridization screening from a meiosis-enriched tassel cDNA library (library 11, inbred line W23, a gift from J. M. Gardiner, University of Arizona, Tucson). The library was screened with a PCR product from maize B73 genomic DNA. Maize B73 genomic DNA from leaf tissue was isolated as previously described [77] with slight modifications: The 2-mercaptoethanol was replaced with 3 mM dithiothreitol (DTT), homogenized tissue samples were incubated at 65°C for 20 min, and the aqueous extraction buffer was supplemented with 1% polyvinylpyrrolidone (Sigma P-5288) and 1% W:V polyvinyl polypyrrolidone (Sigma P-6755). Genomic DNA (20 ng) was used in a 20-μL PCR reaction with forward and reverse *ZmSUN3*/*4*-specific primers (cg1pf1, 5'-GTGATTTGGAGATGCCAGGTG-3' and cg1pr1, 5'-TTTGAGCAAGTTTTGCATTCG-3', respectively) to produce a 639-bp fragment corresponding to a region within exon 2. The PCR product was resolved on 1% agarose, gel purified, and then cloned into the PCR 2.1-TOPO cloning vector (Invitrogen). The plasmid, pSPM17-2, was digested with *Eco*RI, and the insert was gel purified, quantified, and used in a random-primed labeling reaction with α-32p-dCTP (Amersham Rediprime™ II DNA Labeling System) for use in cDNA library screening. Approximately 5 × 10^5 ^phage at a high stringency (Tm-15°C) were screened.

### Antibody Production and Immunoblotting

Amino acids 244-256 (ZmSUN3) were chosen as an epitope for the production of rabbit polyclonal antisera to be used to study PM3 proteins in maize. We selected the sequence (zms3gsp1a, LDKDKDKYLRNPC) to allow for the detection of either of the closely related ZmSUN3 and ZmSUN4 proteins. A second peptide antibody was also generated against ZmSUN3 (zms3gsp2, ENKKTEPDDKTKEP). Antibody production, including synthesis of the peptides and affinity purification, was carried out by GenScript (complete affinity-purified rabbit polyclonal antibody package, SC1031, GenScript Corporation, Piscataway, NJ).

Total maize protein extracts were obtained as previously described [78], with slight modifications: Briefly, one gram of tissue was harvested, ground to a powder in liquid nitrogen, and then homogenized in 3 mL of extraction buffer containing 50 mM Tris-HCl (8.0), 1 mm EDTA-NaOH (8.0), 10% w:v sucrose, 100 mM dithiothreitol, and 1× protease inhibitor complex (4-(2-aminoethyl) benzenesulfonyl fluoride, bestatin, pepstatinA, E-64, leupeptin, and 1,10-phenanthroline, Sigma Aldrich). The homogenate was centrifuged at 12,000 × g for 20 min at 4°C, and the supernatant was recovered and used immediately for immunoblotting or stored at -80°C. For western analyses, protein extracts were mixed with 5× sodium dodecyl sulfate (SDS) loading buffer (25 mM Tris-HCl [6.8], 0.1 M 2*-*mercaptoethanol, 10% SDS, and 50% glycerol), boiled for 5 min, and separated by electrophoresis on a 10% (w/v) SDS-polyacrylamide gel. Proteins were transferred by electroblotting (overnight, 4°C, 30 mA) to a 0.45-μm polyvinylidene fluoride transfer membrane (PALL life sciences, Port Washington, NY) in a Bio-Rad Mini-PROTEAN 3 Cell. After the membranes were blocked with 5% (w/v) nonfat milk in phosphate-buffered saline plus 0.05% [v/v] Tween-20 (PBS-T) buffer, they were incubated with α-zms3gsp1a diluted 1:2,000 with PBS-T at room temperature for 1 h. After four 15-min washes in PBS-T buffer at room temperature, the membranes were incubated with a 1:5,000 dilution (in PBS-T buffer) of anti-rabbit IgG horseradish peroxidase-linked antibody (Santa Cruz Biotechnology, Santa Cruz, CA) for 1 h at room temperature, then subjected to four 15-min washes in PBS-T buffer at room temperature. The immune complexes were visualized with a chemiluminescent reaction kit for 5 min at room temperature (Millipore, Immobilon detection kit, WBKL50100, Billerica, MA).

### Protein Immunolocalization and Microscopy

Maize pollen mother cells were microdissected and fixed in meiocyte Buffer A [45] with 1% paraformaldehyde supplemented with 100 mM DTT for 30 min at room temperature. The anthers were then rinsed in Buffer A alone for 30 min at room temperature and stored at 4°C. Cells were prepared for immunofluorescence microscopy by embedding in polyacrylamide, followed by a 1-h room-temperature treatment in permeabilization buffer (1% Triton X-100, 1 mM EDTA-NaOH, and 1% BSA in 1× PBS). The acrylamide pads on the slides were then incubated in blocking buffer (3% BSA, 5% normal sheep serum, 1 mM EDTA-NaOH, 0.1% Tween-20, and 1 mM DTT in 1× PBS) at 30°C for 2 h and then incubated with the primary antibodies (α-zmS3gsp1a, α-zmS3gsp2, or preimmune sera at 1:50) in blocking buffer or blocking buffer alone (for secondary-only control) overnight at 30°C. After four consecutive 15-min washes at room temperature with 1× PBS, cells were incubated with a FITC-conjugated goat anti-rabbit IgG (1:1500 in blocking buffer) for 1 h at 30°C then given four 15-min washes with 1× PBS at room temperature. Cells were stained with 3 μg/mL DAPI (4',6-diamidino-2-phenylindole) in 1× PBS for 30 min at room temperature, rinsed three times with 1× PBS, treated with vectashield antifading solution, and finally sealed with a 22 × 30 × 1.5 mm coverslip. Images were collected on an Olympus IX-70 epifluorescense microscope, deconvolved, and analyzed with the SoftWorx computerized workstation.

## Authors' contributions

SPM and HWB carried out the bioinformatic analyses. SPM carried out the molecular cloning and immunolocalization experiments. CRS performed the Solexa mRNA transcription profiles for *ZmSUN1-5 *and *GAPDH*. SPM and HWB and CRS interpreted the results, and SPM and HWB wrote the manuscript. All authors read and approved the final manuscript.

## Supplementary Material

Additional file 1 **Multiple sequence alignment of full-length maize CCSD proteins**. Full-length plant CCSD-type protein sequences predicted from cDNAs from maize, sorghum, rice, *Arabidopsis*, and moss were aligned by the maximum-likelihood approach (ClustalW2). Residues with at least 50% similarity are shaded in grey, identical amino acids in black. Click here for file

Additional file 2 **Multiple sequence alignment of full-length maize PM3 proteins**. Full-length plant PM3-type protein sequences predicted from cDNAs from maize, sorghum, rice, *Arabidopsis*, and moss were aligned by the maximum-likelihood approach (ClustalW2). Residues with at least 50% similarity are shaded in grey, identical amino acids in black. Click here for file

Additional file 3 **Gene expression profiles of the maize SUN-domain protein genes available from NCBI's Unigene**. Gene expression data for *ZmSUN1, 2*, and 4 as well as cytoplasmic *GAPDH *are shown. Tissues pooled for each gene are indicated at the left, and the corresponding Unigene accession numbers are indicated for each gene. Click here for file

Additional file 4 **Solexa expression data for B73 ZmSUN genes**. Expression data are given here as transcripts per ten million for each of the maize *ZmSUN *genes. Platforms, sample ID's, tissue, and developmental stages are also given. WT = Solexa whole transcriptome; Tag = Solexa tag-based. Click here for file
