# Supplementary material for: Structure and expression of the maize (Zea mays L.) SUN-domain protein gene family: evidence for the existence of two divergent classes of SUN proteins in plants
Source: BMC Plant Biol. 2010 Dec 8;10:269. doi: 10.1186/1471-2229-10-269 (PMC3017857; doi:10.1186/1471-2229-10-269)
Supplement: Additional file 2 — Multiple sequence alignment of full-length maize PM3 proteins. Full-length plant PM3-type protein sequences predicted from cDNAs from maize, sorghum, rice, Arabidopsis, and moss were aligned by the maximum-likelihood approach (ClustalW2). Residues with at least 50% similarity are shaded in grey, identical amino acids in black. [file 1471-2229-10-269-S2.PDF]

```
ZmSUN3        ------------------------MQRSRRALLRRTAAAQEQSAVAEAAA--NGRRRRLYGFSASLVVASWVAVLLLHSL
ZmSUN4        MSLSCWRVRFPGADVREAGRGREGMQRSRKALLRRTAAAQVQSAVAEAAG--NGRKRRLYGFSVSLVVTLWVAVLLLHSL
ZmSUN5        --------------MSRKRREGGGGGRGAGP--VDHHGGGKGSEAGAAATDAVSMDGGLREVSVPVVFSVWCLLFLLRSQ
Sb03g026980   --------------MSRKRREGGGGGRGAGTGTGDHHGGGSGKGSGAGA-DAVSMDGGLREVSVSVVFSVWCILFLLRSQ
Sb03g041510   ------------------------MQRSRRALLRRTAAAQEQSAVAEAEAAANGRKRRLYGFSASLVVASWVAVLLLHSL
Os01g41600    ------------------------------------------------------MDGGLREVSLSVVFSVWCLLFLLRSQ
At1g71360     ------------------------MQRSRRALLVRRRVSETTS---------NGRNR-FYKVSLSLVFLIWGLVFLSTLW
At1g22882     ------------------------MQRSCR---TRRRVSVNKF---------NGRNS-FYKVSLSLVFLLWVLLFFSTLL
XP_001775438  ----------MQKKGGKSKHHRAAGKEVERIERCEGVALFQKDLQQQQLQELGKERKGCYRISWCAVVSISATLFLFLSL
XP_001758570  ----------MLKKVAKSKHLKATRKKSERIERCKGDQLSQKELLQP---EHGRRRYGSYGISWFAVARIPAALLVFVPL
consensus                                                                   *   *
              1.......10........20........30........40........50........60........70........80

ZmSUN3        VGHGDGQRD-------------------------------------------GGGSTVDLTVVEPTVNVGPINPVVQEEH
ZmSUN4        VGHGDGQRD-------------------------------------------GGGSGVDITFIEPALNGGPVNSAVQEVH
ZmSUN5        FLHSQADDD-------------------------------------------------PSSEFYEEH--GMRDSYCKVRP
Sb03g026980   FLHSQTDDD-------------------------------------------------PSSEFYEDHH-GRRDSYCKVRP
Sb03g041510   VGHGDSQRD-------------------------------------------GGGYAVDLTVVEPALNVGPFNPVVQVEH
Os01g41600    FLHSQTD----------------------------------------------------PSDFYDDVEDGMRENYCKVMP
At1g71360     ISHVDGDKGR------------------------------------------SLVDSVEKGEPDDERADETAESVD-ATS
At1g22882     ISHGDGAKDE------------------------------------------PLNDSMGMADPDDGQSDEKVVPFDGPLS
XP_001775438  FPSLPSIVKLPDLEGVRASWQHFSLYSRLSHP-------EFEISGLSHFTPLVSKNWVSPFSLSKTKELHSRKWFASQSS
XP_001758570  VLLFPSIEKLPNLEVIQPDRVIARLVGGINVVNGVSVLVLGELVGFFHSTILAARNRAAPSSLSKTRESHSKKSYASHLS
consensus
              ........90.......100.......110.......120.......130.......140.......150.......160

ZmSUN3        GGDLEMPGDSCVNSDENAVLSEDTLVQADQ------------------LCSSDEVQNENTEAVTIDS-------------
ZmSUN4        GENLAVPSDTCVGSVENAVLPEDTLVQAAQ------------------LCSNDEARSENTEALTKNN-------------
ZmSUN5        LEAYVLP--YHNDS----CQSSYSHSQPPQ--------------------------------------------------
Sb03g026980   LEAYVLP--YHNDSSTTTCQSSYSQPQPPQ--------------------------------------------------
Sb03g041510   GENLAVPGDPCVNSVENAVLSEDTLVQADQ------------------LCSNDEVLSENTEALTKDS-------------
Os01g41600    LEAYIFPTEYNASAAAPTCQPSLHPPDQPQ--------------------------------------------------
At1g71360     LESTSVHSNPGLSSDVDIAAAGESKGSET-------------------ILKQLEVDN--TIVIVG---------------
At1g22882     LASASVDVTSDLSRNDDVNLSEESEDKEQEAEISSTVSGNDIESKDTYLLKQSEINKKDTGIDAGSKYDDFPKKSEINNT
XP_001775438  GQRCHLNDTAHLDADTCLIFNGSAVIGNGG----CLKSIYAGDGSRNSHESFSASRKYMSFFASASSVAEEADHFLPVES
XP_001758570  RQWCYLNDTEHLDAGACSVVNGSMAFANGDTQSLGTKIIHTIEG-KSLYEELSASKKHMSFFASASSVAEDGHDFFPVES
consensus
              ........170.......180.......190.......200.......210.......220.......230.......240

ZmSUN3        -----------------------------------------QVELSGDQGGYLPQ-------------------------
ZmSUN4        -----------------------------------------QVELSGDQCGYLPQ-------------------------
ZmSUN5        --------------------------------------------EAPSSSALAS--------------------------
Sb03g026980   --------------------------------------------ESPSASAPAPPE------------------------
Sb03g041510   -----------------------------------------QVELSGDQGGYLPQ-------------------------
Os01g41600    --------------------------------------------QETDHRSLEP--------------------------
At1g71360     -----------------------------------------NVTESKD-NVPMKQ-------------------------
At1g22882     --------------------------------------GTWNDTEGKDDNNFLKQ-------------------------
XP_001775438  PSPSSSDEESSSLGDVIISLPWSSSVGEPLKLCLVGETGADCLRHSSDGDAFSSQ----GSPDGTVSWVSELPSLDQICP
XP_001758570  -AAFIADEEAPSRRNGATSLPWSSGLRQPLKFCLVGVAGVDCLRHSSESDASTVQLLEHGSPNGTISWLSELPFLDQMCC
consensus
              ........250.......260.......270.......280.......290.......300.......310.......320

ZmSUN3        ------------------SGVD---------------------SGVQPGEKVESKDLPRPPRLSRVAPPDLDEFKTRAIA
ZmSUN4        ------------------PDFD---------------------SGVQPGEKVESEDLPRPPRLSRVAPPDLDEFKTRAIA
ZmSUN5        -----------------------------------------------PQYNATTGG--NASSPE-AAFVGLDEFRSRMMQ
Sb03g026980   -----------------LP----------------------------PQYNATTGGGNNASSPEAAAFVGLDEFRSRIMQ
Sb03g041510   ------------------SDVD---------------------SGVQPGEKVESEDLPRPPRLSRVAPPDLDEFKTRAIA
Os01g41600    -------------------------------------------------FNNTTGG---KSSAEAAALDELDEFRSRILQ
At1g71360     ------------------SEIN---------------------NNTVPGNDTETT-GSKLDQLSRAVPLGLDEFKSRASN
At1g22882     ------------------SQLNKTGTGNDTESSDNEFLEQNQMNKTVLGNGTEIN-VSKVDQPSRAVPLGLDEFKSRASN
XP_001775438  TQESLPSSSDSEAELSSAIQINQQYLVCRRNLGTGSVVCEPLCQVSTLDYLTQSAARVCPCSPSLVEESNADASRISFSG
XP_001758570  SRERLSTSGDSE-----AIVIDKPCLICQRDSLTDSVSCEPLCQMPTINHLTQSATRVCLCGPSFVEAFSADVSKFRLSG
consensus                                                                                *
              ........330.......340.......350.......360.......370.......380.......390.......400

ZmSUN3        ERGPGISS----QPGNIIHRREPS--------------------------------------------------------
ZmSUN4        ERGPGISS----QPGNVVHRREPS--------------------------------------------------------
ZmSUN5        GKAENDTG--PPTDGGVAHRLEPN--------------------------------------------------------
Sb03g026980   GKAENDTGRPRPTDGGAAHRLEPN--------------------------------------------------------
Sb03g041510   ERGPGVSS----QPGHVIHRREPS--------------------------------------------------------
Os01g41600    GKAENGRVPDGATP--AAHRLEPS--------------------------------------------------------
At1g71360     SRDKSLSG----QVTGVIHRMEPG--------------------------------------------------------
At1g22882     SRNKSLSD----QVSGVIHRMEPG--------------------------------------------------------
XP_001775438  SLLSKASSVDELQISDAPSLEEPSEVPEISFDIVTQTLANEDVSEEIVKFDELVEIQQEVSRPSRVVQVKSLDEYKKAVI
XP_001758570  NPFNTTPS-GEAETTETPSLGQPSEVPIVPLEIVTQSSADQAVKEELLKSDTSVEVQQELSRPLRVTQVKSLDEYKKTVL
consensus                                *
              ........410.......420.......430.......440.......450.......460.......470.......480
```

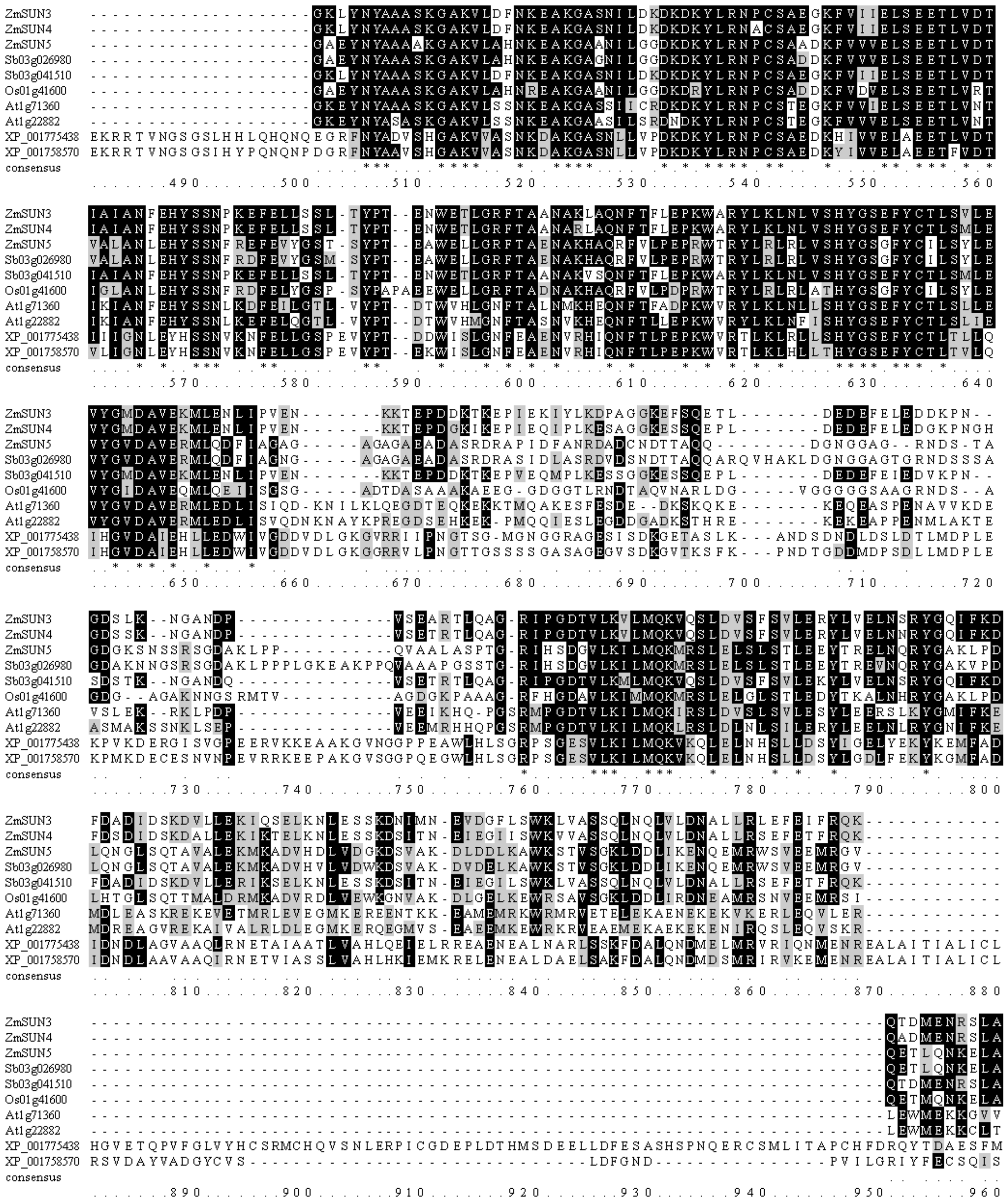
ZmSUN3
ZmSUN4
ZmSUN5
Sb03g026980
Sb03g041510
Os01g41600
At1g71360
At1g22882
XP_001775438
XP_001758570
consensus

```
ZmSUN3       VIFLSFVFACLALAKLSIG-------------------IMSRFCRFYN------FEKFHNVRSGWLVLLLSSCVISTILI
ZmSUN4       VIFLSFVFACLALAKLSIG-------------------IMSRFCRFYD------FEKFHNVRSGWLVLLLSSCIISTILI
ZmSUN5       VLSISLFFACLALFKLACD-------------------RVFCLFAGKG-REEPDAEEHTRSSRAWMLVLASSSFTTLIVL
Sb03g026980  VLSISLFFACLALFKLACD-------------------RLLCLFAGKGSREEADAEEHTRSSRAWMLVLASSSFTTLIVL
Sb03g041510  VIFLSFVFACLALAKLSIG-------------------IMSKFCRFYD------FEKIHNVRSGWVVLLLSSCIISTILI
Os01g41600   VLSISLFFACLALFKLACD-------------------RVLFLFTRKG---AAAAERMCGASKGWILVLASSSFTTFLVL
At1g71360    VFTICVGFGTIAVVAVVFG-------------------MGIVRA-----------EKQG--GLAWLLLLISSTFVMFILS
At1g22882    VFTVCLGFGIIAVIAVVIG-------------------MGTGLA-----------EKTG--SGAWLLLLISSTFIMFVLS
XP_001775438 NLSVVSCQQLSALMCYALSSSKIEVDPFLPTCKYLT--VLNIYLNRRTSRRAPDPATTRKPYVQVTLVLCCSNFTHVKRN
XP_001758570 YTRTIEFRWFCALISFHME---------VPDRHYLEGAVVRILLN-------PD-ANQQQSCRFLVFSECPCNFSLSSLK
consensus    ...........*.......                   ..  .          ..       ...............
             ......970......980......990.....1000.....1010.....1020.....1030.....1040

ZmSUN3       IQ------------------------------------------------------------------------------
ZmSUN4       IQ------------------------------------------------------------------------------
ZmSUN5       LYN-----------------------------------------------------------------------------
Sb03g026980  LYN-----------------------------------------------------------------------------
Sb03g041510  IQ------------------------------------------------------------------------------
Os01g41600   LYN-----------------------------------------------------------------------------
At1g71360    L-------------------------------------------------------------------------------
At1g22882    L-------------------------------------------------------------------------------
XP_001775438 VLEVWSVPTSLSFQIEASHLLRWRSHYAGVPQTTISEPSSVTLEYTHYDWSSLIDSWTWRYRVQRFVRAEYRRFGQGESH
XP_001758570 LLDLLEVR------------------------------------------------------------------------
consensus    .
             .....1050.....1060.....1070.....1080.....1090.....1100.....1110.....1120

ZmSUN3       --------------------------------------------------------------------------------
ZmSUN4       --------------------------------------------------------------------------------
ZmSUN5       --------------------------------------------------------------------------------
Sb03g026980  --------------------------------------------------------------------------------
Sb03g041510  --------------------------------------------------------------------------------
Os01g41600   --------------------------------------------------------------------------------
At1g71360    --------------------------------------------------------------------------------
At1g22882    --------------------------------------------------------------------------------
XP_001775438 TRMSQRRQSHPIWCCMCPTVLVKPARGCSDENEAMEIGETLSSIELSSSGTAMALKTRFYLWLCSFMLLGNCLLHTVEGG
XP_001758570 ----------------------------------------LSSIELSSAGTAMALKTRFYLWLCSFVLFVNCLLYTVDGG
consensus
             .....1130.....1140.....1150.....1160.....1170.....1180.....1190.....1200

ZmSUN3       --------------------------------------------------------------------------------
ZmSUN4       --------------------------------------------------------------------------------
ZmSUN5       --------------------------------------------------------------------------------
Sb03g026980  --------------------------------------------------------------------------------
Sb03g041510  --------------------------------------------------------------------------------
Os01g41600   --------------------------------------------------------------------------------
At1g71360    --------------------------------------------------------------------------------
At1g22882    --------------------------------------------------------------------------------
XP_001775438 ELGLGLTAAVSVKHPQTYVNRKLRASTPSCTKADISITQGKSGNSNGIPAFSVQITNLCVNHNCQLKNIHVACAAFASAR
XP_001758570 ELTLGVAAAFSVKHPQTYVNRKLRASIPSCTKADISITQGKSGNSNGIPAFSVQITNLCINHNCQLRNIHVACAAFASAR
consensus
             .....1210.....1220.....1230.....1240.....1250.....1260.....1270.....1280

ZmSUN3       ----------------------------------------------------
ZmSUN4       ----------------------------------------------------
ZmSUN5       ----------------------------------------------------
Sb03g026980  ----------------------------------------------------
Sb03g041510  ----------------------------------------------------
Os01g41600   ----------------------------------------------------
At1g71360    ----------------------------------------------------
At1g22882    ----------------------------------------------------
XP_001775438 PLDSHVFQRIKYNDCLVMGGAPLRAGGSVAFEYANSSEYPMHVISAELGPCA
XP_001758570 PLDSRVFQRIKYNDCLVMGGAPLRAGGSVAFEYANSSEYPMHVISADLGPCS
consensus
             .....1290.....1300.....1310.....1320.....1330..
```
