# Supplementary material for: Structure and expression of the maize (Zea mays L.) SUN-domain protein gene family: evidence for the existence of two divergent classes of SUN proteins in plants
Source: BMC Plant Biol. 2010 Dec 8;10:269. doi: 10.1186/1471-2229-10-269 (PMC3017857; doi:10.1186/1471-2229-10-269)
Supplement: Additional file 3 — Gene expression profiles of the maize SUN-domain protein genes available from NCBI's Unigene. Gene expression data for ZmSUN1, 2, and 4 as well as cytoplasmic GAPDH are shown. Tissues pooled for each gene are indicated at the left, and the corresponding Unigene accession numbers are indicated for each gene. [file 1471-2229-10-269-S3.PDF]

Tissue Pool

Gene Expression Profile

Tissue Pool	*ZmSUN1* Zm.94705		*ZmSUN2* Zm.6043		*ZmSUN4* Zm.17612		*ZmGAPDH* Zm.3765	
aerial organ	0	0 / 10002	0	0 / 10002	0	0 / 10002	399	4 / 10002
aleurone	0	0 / 2119	0	0 / 2119	0	0 / 2119	0	0 / 2119
cell culture	0	0 / 13389	0	0 / 13389	0	0 / 13389	970	13 / 13389
cell lignification	0	0 / 1893	0	0 / 1893	0	0 / 1893	0	0 / 1893
ear	222	8 / 35905	139	5 / 35905	0	0 / 35905	1002	36 / 35905
embryo	0	0 / 19891	50	1 / 19891	100	2 / 19891	1005	20 / 19891
endosperm	18	1 / 55328	54	3 / 55328	0	0 / 55328	885	49 / 55328
glume	0	0 / 2177	0	0 / 2177	0	0 / 2177	918	2 / 2177
leaf	0	0 / 14687	0	0 / 14687	0	0 / 14687	1089	16 / 14687
meristem	76	12 / 156203	6	1 / 156203	0	0 / 156203	339	53 / 156203
ovary	0	0 / 22915	43	1 / 22915	0	0 / 22915	218	5 / 22915
pedicel	0	0 / 2904	0	0 / 2904	0	0 / 2904	2066	6 / 2904
pericarp	0	0 / 9593	0	0 / 9593	312	3 / 9593	625	6 / 9593
pollen	58	1 / 17164	174	3 / 17164	0	0 / 17164	116	2 / 17164
root	0	0 / 15670	0	0 / 15670	0	0 / 15670	382	6 / 15670
sheath	0	0 / 2890	0	0 / 2890	0	0 / 2890	1038	3 / 2890
shoot	53	5 / 93902	0	0 / 93902	42	4 / 93902	223	21 / 93902
silk	0	0 / 1544	0	0 / 1544	0	0 / 1544	1295	2 / 1544
tassel	84	2 / 23799	0	0 / 23799	0	0 / 23799	798	19 / 23799

Number of transcripts per million ESTs

Gene EST

Total number of ESTs in pool

Spot intensity
