# Supplementary material for: Structure and expression of the maize (Zea mays L.) SUN-domain protein gene family: evidence for the existence of two divergent classes of SUN proteins in plants
Source: BMC Plant Biol. 2010 Dec 8;10:269. doi: 10.1186/1471-2229-10-269 (PMC3017857; doi:10.1186/1471-2229-10-269)
Supplement: Additional file 4 — Solexa expression data for B73 ZmSUN genes. Expression data are given here as transcripts per ten million for each of the maize ZmSUN genes. Platforms, sample ID's, tissue, and developmental stages are also given. WT = Solexa whole transcriptome; Tag = Solexa tag-based. [file 1471-2229-10-269-S4.PDF]

Supplemental Table1. Solexa Expression Data for ZmSUN Genes in Maize Inbred B73.								
				Expression values in parts per 10 million				
Platform and Sample Number	Sample ID	Dev. Stage	Tissue	ZmSUN1 (GenBank EU964563)	ZmSUN2 (GenBank BT055722)	ZmSUN3 (GRMZM2G1 22 914_T01)	ZmSUN4 (GenBank GU453173)	ZmSUN5 (GenBank EU953247)
WT_001	A0140042	V5	Root	384	203	91	69	0
WT_002	A0140043	V19	Root	205	126	83	37	0
WT_003	A0140066	V5	Leaf	191	118	24	13	0
WT_004	A0140067	V5	Leaf	110	100	30	13	0
WT_005	A0140068	V5	Leaf	99	78	25	13	0
WT_006	A0140040	V5	Leaf	86	106	14	2	0
WT_007	A0140041	V19	Leaf	159	102	25	18	0
WT_008	A0140051	V8	Stalk	191	159	56	39	0
WT_009	A0140069	V8	Immature Ear	492	204	220	67	0
WT_010	A0140070	V8	Immature Ear	266	232	74	40	0
WT_011	A0140071	V8	Immature Ear	223	200	57	58	0
WT_012	A0140044	V8	Immature Ear	287	242	46	44	0
WT_013	A0140045	V19	Immature Ear	143	140	53	66	0
WT_014	A0140049	R4	Embryo	183	70	59	26	0
WT_015	A0140047	R2	Kernel	75	43	36	24	0
WT_016	A0140048	R4	Endosperm	194	142	81	78	0
WT_017	A0140050	R4	Pericarp	159	100	93	46	0
WT_018	A0290001	V5	Tassel	116	102	34	16	3
WT_022	A0290002	V6	Tassel	435	184	95	86	0
WT_026	A0290003	V7	Tassel	444	172	110	81	0
WT_030	A0140046	V19	Tassel	364	200	71	52	0
WT_031	A0140052	R1	Pollen	71	49	0	2	116
Tag_001	A0400001	V5	Root	107	349	38	27	0
Tag_002	A0400002	V5	Root	98	272	99	52	0
Tag_011	A0140007	V5	Root	166	403	158	96	0
Tag_012	A0140008	V5	Root	126	332	79	63	2
Tag_013	A0140009	V5	Root	127	439	222	231	0
Tag_014	A0140010	V19	Root	129	436	147	99	0
Tag_015	A0140011	V19	Root	120	237	82	35	0
Tag_016	A0140012	V19	Root	86	256	23	20	0
Tag_017	A0140001	V5	Leaf	65	362	45	20	0
Tag_018	A0140002	V5	Leaf	112	237	55	21	0
Tag_019	A0140003	V5	Leaf	167	667	131	84	2
Tag_020	A0090019	V14	Leaf	118	435	41	26	0
Tag_021	A0090020	V14	Leaf	117	324	38	30	0
Tag_022	A0090021	V14	Leaf	84	301	32	30	0
Tag_023	A0090022	V14	Leaf	45	178	9	3	0
Tag_024	A0090023	V14	Leaf	69	131	11	6	0
Tag_025	A0090024	V14	Leaf	55	196	16	9	0
Tag_026	A0090025	V14	Leaf	39	228	13	7	0
Tag_027	A0090026	V14	Leaf	43	246	11	6	0
Tag_028	A0090027	V14	Leaf	66	305	36	19	0
Tag_029	A0090028	V14	Leaf	52	209	12	7	0
Tag_030	A0090029	V14	Leaf	77	185	3	3	0
Tag_031	A0090030	V14	Leaf	43	195	10	4	0
Tag_032	A0090031	V14	Leaf	57	208	33	10	0
Tag_033	A0090032	V14	Leaf	46	289	26	3	2

Tag_034	A0090033	V14	Leaf	28	211	13	2	0
Tag_035	A0090034	V14	Leaf	36	264	5	0	0
Tag_036	A0090035	V14	Leaf	70	202	19	6	0
Tag_037	A0090036	V14	Leaf	62	194	11	2	0
Tag_038	A0140004	V19	Leaf	44	227	36	20	0
Tag_039	A0140005	V19	Leaf	45	267	24	9	0
Tag_040	A0140006	V19	Leaf	57	243	28	10	0
Tag_041	A0140034	V8	Stalk	83	332	131	71	1
Tag_042	A0140035	V8	Stalk	167	364	166	108	0
Tag_043	A0140036	V8	Stalk	203	321	164	102	0
Tag_044	A0140013	V8	Immature Ear	252	730	138	80	0
Tag_045	A0140015	V8	Immature Ear	151	1163	134	89	0
Tag_046	A0220001	V10	Immature Ear	184	309	132	100	0
Tag_047	A0220002	V10	Immature Ear	90	498	131	63	0
Tag_048	A0220003	V10	Immature Ear	48	298	47	23	0
Tag_052	A0090001	V14	Immature Ear	152	680	132	50	0
Tag_053	A0090002	V14	Immature Ear	116	791	111	59	2
Tag_054	A0090003	V14	Immature Ear	154	662	98	54	0
Tag_055	A0090004	V14	Immature Ear	154	639	206	88	0
Tag_056	A0090005	V14	Immature Ear	180	611	123	54	0
Tag_057	A0090006	V14	Immature Ear	201	544	205	60	0
Tag_058	A0090007	V14	Immature Ear	151	645	130	48	0
Tag_059	A0090008	V14	Immature Ear	140	551	127	53	0
Tag_060	A0090009	V14	Immature Ear	142	662	115	61	0
Tag_061	A0090010	V14	Immature Ear	158	805	89	36	0
Tag_062	A0090012	V14	Immature Ear	109	586	127	62	0
Tag_063	A0090013	V14	Immature Ear	131	730	92	35	2
Tag_064	A0090014	V14	Immature Ear	142	734	148	71	0
Tag_065	A0090015	V14	Immature Ear	182	613	138	68	0
Tag_066	A0090016	V14	Immature Ear	154	654	149	48	0
Tag_067	A0090017	V14	Immature Ear	137	832	127	52	0
Tag_068	A0090018	V14	Immature Ear	127	706	102	35	0
Tag_069	A0140016	V19	Immature Ear	186	722	139	64	0
Tag_070	A0140017	V19	Immature Ear	219	794	125	72	0
Tag_071	A0140018	V19	Immature Ear	135	760	181	87	0
Tag_072	A0140028	R4	Embryo	67	195	70	30	0
Tag_073	A0140029	R4	Embryo	77	195	56	31	0
Tag_074	A0140030	R4	Embryo	47	156	47	14	0
Tag_075	A0140022	R2	Kernel	43	172	108	43	0
Tag_076	A0140023	R2	Kernel	33	177	104	31	0
Tag_077	A0140024	R2	Kernel	34	101	47	22	0
Tag_078	A0140025	R4	Endosperm	90	376	195	66	0
Tag_079	A0140026	R4	Endosperm	65	384	238	101	0
Tag_080	A0140027	R4	Endosperm	81	500	206	93	0
Tag_081	A0140031	R4	Pericarp	39	344	146	59	0
Tag_082	A0140032	R4	Pericarp	60	309	147	58	0
Tag_083	A0140033	R4	Pericarp	82	256	180	101	0
Tag_084	A0140019	V19	Tassel	41	274	40	15	0
Tag_085	A0140020	V19	Tassel	44	224	44	12	3
Tag_086	A0140021	V19	Tassel	112	288	218	65	0
Tag_087	A0140037	R1	Pollen	68	110	50	14	35
Tag_088	A0140038	R1	Pollen	41	131	29	11	28
Tag_089	A0140039	R1	Pollen	66	131	85	24	49

Footnotes:

Platform: WT = Solexa Whole Transcriptome; Tag = Solexa Tag-based

Developmental stage designations are as described for Figure 6.

Accession numbers for corresponding genes are shown in parentheses and are from B73, except for ZmSUN4.

The ZmSUN3 sequence is a gene model from www.maizeseqence.org, B73 AGPv1, 2010
