# Supplementary material for: Structure and expression of the maize (Zea mays L.) SUN-domain protein gene family: evidence for the existence of two divergent classes of SUN proteins in plants
Source: BMC Plant Biol. 2010 Dec 8;10:269. doi: 10.1186/1471-2229-10-269 (PMC3017857; doi:10.1186/1471-2229-10-269)
Supplement: Additional file 1 — Multiple sequence alignment of full-length maize CCSD proteins. Full-length plant CCSD-type protein sequences predicted from cDNAs from maize, sorghum, rice, Arabidopsis, and moss were aligned by the maximum-likelihood approach (ClustalW2). Residues with at least 50% similarity are shaded in grey, identical amino acids in black. [file 1471-2229-10-269-S1.PDF]

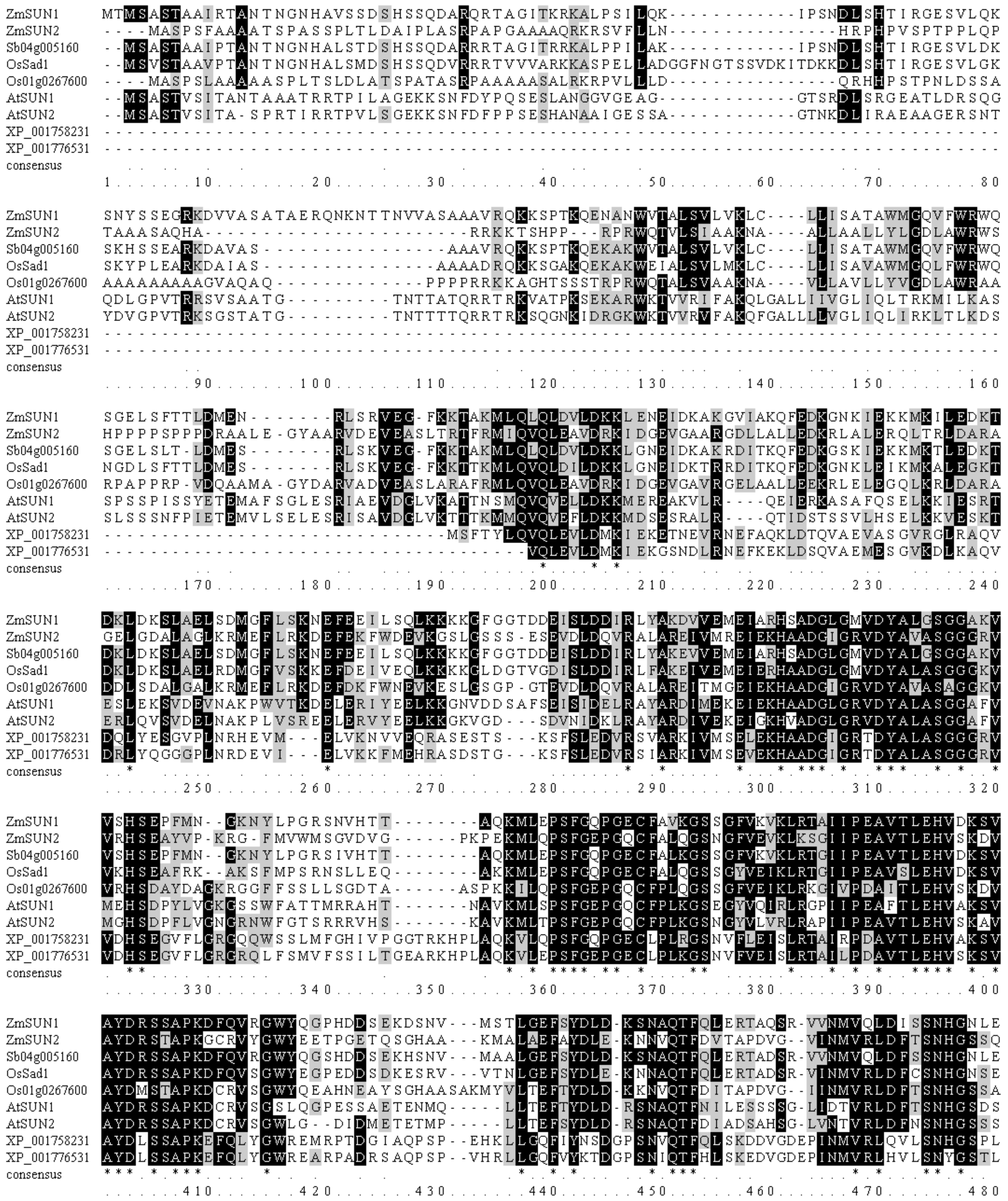
ZmSUN1 MTMSASTAAIRTANTNGNHAVSSDSHSSQDARQRTAGITKRKALPSILQK------------IPSNDLSHTIRGESVLQK
ZmSUN2 ----MASPSFAAAATSPASSPLTLDAIPLASRPAPGAAAAQRKRSVFLLN----------------HRPHPVSPTPPLQP
Sb04g005160 --MSASTAAIPTANTNGNHALSTDSHSSQDARRRTAGITRRKALPPILAK------------IPSNDLSHTIRGESVLDK
OsSad1 --MSVSTAAVPTANTNGNHALSMDSHSSQDVRRRTVVVARKKASPELLADGGFNGTSSVDKITDKKDLSHTIRGESVLGK
Os01g0267600 ----MASPSLAAAAASPLTSLDLATSPATASRPAAAAASALRKRPVLLLD----------------QRHHPSTPNLDSSA
AtSUN1 --MSASTVSITANTAAATRRTPILAGEKKSNFDYPQSESLANGGVGEAG-------------GTSRDLSRGEATLDRSQG
AtSUN2 --MSASTVSITA-SPRTIRRTPVLSGEKKSNFDFPPSESHANAAIGESSA------------GTNKDLIRAEAAGERSNT
XP_001758231 --
XP_001776531 --
consensus
ZmSUN1 SNYSSEGRKDVVASATAERQNKNTTNVVASAAAVRQKKSPTKQENANWVTALSVLVKLC----LLISATAWMGQVFWRWQ
ZmSUN2 TAAASAQHA-----------------------RRKKTSHPP---RPRWQTVLSIAAKNA----ALLAALLYLGDLAWRWS
Sb04g005160 SKHSSEARKDAVAS----------------AAAVRQKKSPTKQEKAKWVTALSVLVKLC----LLISATLWMGQVFWRWS
OsSad1 SKYPLEARKDAIAS----------------AAADRQKKSGAKQEKAKWEIALSVLMKLC----LLISAVAWMGQLFWRWQ
Os01g0267600 AAAAAAAAAGVAQAQ-------------PPPPRRKKAGHTSSSTRPRWQTALSVAAKNA----VLLAVLLYVGDLAWRAA
AtSUN1 QDLGPVTRRSVSAATG---------TNTTATQRRTRKVATPKSEKARWKTVVRIFAKQLGALLIIVGLIQLTRKMILKAS
AtSUN2 YDVGPVTRKSGSTATG---------TNTTTTQRRTRKSQGNKIDRGKWKTVVRVFAKQFGALLLLVGLIQLIRKLTLKDS
XP_001758231 --
XP_001776531 --
consensus
ZmSUN1 SGELSFTTLDMEN-------RLSRVEG-FKKTAKMLQLQLDVLDKKLENEIDKAKGVIAKQFEDKGNKIEKKMKILEDKT
ZmSUN2 HPPPPSPPPDRAALE-GYAARVDEVEASLTRTFRMIQVQLEAVDRKIDGEVGAARGDLLALLEDKRLALERQLTRLDARA
Sb04g005160 SGELSLT-LDMES-------RLSKVEG-FKKTAKMLQLQLDVLDKKLGNEIDKAKRDITKQFEDKGSKIEKKMKTLEDKT
OsSad1 NGDLSFTTLDMES-------RLSKVEG-FKKTTKMLQVQLDILDKKLGNEIDKTRRDITKQFEDKGNKLEIKMKALEGKT
Os01g0267600 RPAPPRP-VDQAAMA-GYDARVADVEASLARAFRMLQVQLEAVDRKIDGEVGAVRGELAALLEEKRLELEGQLKRLDARA
AtSUN1 SPSSPISSYETEMAFSGLESRIAEVDGLVKATTNSMQVQVELLDKKMEREAKVLR----QEIERKASAFQSELKKIESRT
AtSUN2 SLSSSNFPIETEMVLSELESRISAVDGLVKTTTKMMQVQVEFLDKKMDSESRALR----QTIDSTSSVLHSELKKVESKT
XP_001758231 ------------------------------MSFTYLQVQLEVLDMKIEKETNEVRNEFAQKLDTQVAEVASGVRGLRAQV
XP_001776531 -------------------------------------VQLEVLDMKIEKGSNDLRNEFKEKLDSQVAEMESGVKDLKAQV
consensus
ZmSUN1 DKLDKSLAELSDMGFLSKNEFEEILSQLKKKKGFGGTDDEISLDDIRLYAKDVVEMEIARHSADGLGMVDYALGSGGAKV
ZmSUN2 GELGDALAGLKRMEFLRKDEFEKFWDEVKGSLGSSS-ESEVDLDQVRALAREIVMREIEKHAADGIGRVDYAVASGGGRV
Sb04g005160 DKLDKSLAELSDMGFLSKNEFEEILSQLKKKKGFGGTDDEISLDDIRLYAKEVVEMEIARHSADGLGMVDYALGSGGAKV
OsSad1 DKLDKSLAELRDMGFVSKKEFDEIVEQLKKKKGLDGTVGDISLDDIRLFAKEIVEMEIERHAADGLGMVDYALASGGGKV
Os01g0267600 DDLSDALGALKRMEFLRKDEFDKFWNEVKESLGSGP-GTEVDLDQVRALAREITMGEIEKHAADGIGRVDYAVASAGGKV
AtSUN1 ESLEKSVDEVNAKPWVTKDELERIYEELKKGNVDDSAFSEISIDELRAYARDIMEKEIEKHAADGLGRVDYALASGGAFV
AtSUN2 ERLQVSVDELNAKPLVSREELERVYEELKKGKVGD---SDVNIDKLRAYARDIVEKEIGKHVADGLGRVDYALASGGAFV
XP_001758231 DQLYESGVPLNRHEVM---ELVKNVVEQRASESTS---KSFSLEDVRSVARKIVMSELEKHAADGIGRTDYALASGGGRV
XP_001776531 DRLYQGGGPLNRDEVI---ELVKKFMEHRASDSTG---KSFSLEDVRSIARKIVMSEVEKHAADGIGRTDYALASGGGRV
consensus
ZmSUN1 VSHSEPFMN--GKNYLPGRSNVHTT--------AQKMLEPSFGQPGECFAVKGSSGFVKVKLRTAIIPEAVTLEHVDKSV
ZmSUN2 VRHSEAYVP-KRG-FMVWMSGVDVG------PKPEKMLQPSFGEPGQCFALQGSNGFVEVKLKSGIIPEAVTLEHVSKSV
Sb04g005160 VSHSEPFMN--GKNYLPGRSIVHTT--------AQKMLEPSFGQPGECFALKGSSGFVKVKLRTGIIPEAVTLEHVDKSV
OsSad1 VKHSEAFRK--AKSFMPSRNSLLEQ--------AKKMLEPSFGQPGECFALQGSSGYVEIKLRTGIIPEAVSLEHVDKSV
Os01g0267600 VRHSDAYDAGKRGGFFSSLLSGDTA------ASPKKILQPSFGEPGQCFPLQGSSGFVEIKLRKGIVPDAITLEHVSKDV
AtSUN1 MEHSDPYLVGKGSSWFATTMRRAHT-------NAVKMLSPSFGEPGQCFPLKGSEGYVQIRLRGPIIPEAFTLEHVAKSV
AtSUN2 MGHSDPFLVGNGRNWFGTSRRRVHS-------KAVKMLTPSFGEPGQCFPLKGSNGYVLVRLRAPIIPEAVTLEHVSKAV
XP_001758231 VDHSEGVFLGRGQQWSSLMFGHIVPGGTRKHPLAQKVLQPSFGQPGECLPLRGSNVFLEISLRTAIRPDAVTLEHVAKSV
XP_001776531 VDHSEGVFLGRGRQLFSMVFSSILTGEARKHPLAQKVLEPSFGEPGECLPLKGSNVFVEISLRTAILPDAVTLEHVSKSV
consensus
ZmSUN1 AYDRSSAPKDFQVRGWYQGPHDDSEKDSNV---MSTLGEFSYDLD-KSNAQTFQLERTAQSR-VVNMVQLDISSNHGNLE
ZmSUN2 AYDRSTAPKGCRVYGWYEETPGETQSGHAA---KMALAEFAYDLE-KNNVQTFDVTAPDVG--VINMVRLDFTSNHGSSQ
Sb04g005160 AYDRSSAPKDFQVRGWYQGSHDDSEKHSNV---MAALGEFSYDLD-KSNAQTFQLERTADSR-VVNMVQLDFSSNHGNLE
OsSad1 AYDRSSAPKDFQVSGWYEGPEDDSDKESRV---VTNLGEFSYDLE-KNNAQTFQLERTADSR-VINMVRLDFCSNHGNSE
Os01g0267600 AYDMSTAPKDCRVSGWYQEAHNEAYSGHAASAKMYVLTEFTYDLD-KKNVQTFDITAPDVG--IINMVRLDFTSNHGSSA
AtSUN1 AYDRSSAPKDCRVSGSLQGPESSAETENMQ-----LLTEFTYDLD-RSNAQTFNILESSSSG-LIDTVRLDFTSNHGSDS
AtSUN2 AYDRSSAPKDCRVSGWLG--DIDMETETMP-----LLTEFSYDLD-RSNAQTFDIADSAHSG-LVNTVRLDFNSNHGSSS
XP_001758231 AYDLSSAPKEFQLYGWREMRPTDGIAQPSP--EHKLLGQFIYNSDGPSNVQTFQLSKDDVGDEPINMVRLQVLSNHGSPL
XP_001776531 AYDLSSAPKEFQLYGWREARPADRSAQPSP--VHRLLGQFVYKTDGPSNIQTFHLSKEDVGDEPINMVRLHVLSNYGSTL
consensus

```
ZmSUN1        VSHSEPFMN--GKNYLPGRSNVHTT--------AQKMLEPSFGQPGECFAVKGSSGFVKVKLRTAIIPEAVTLEHVDKSV
ZmSUN2        VRHSEAYVP-KRG-FMVWMSGVDVG------PKPEKMLQPSFGEPGQCFALQGSNGFVEVKLKSGIIPEAVTLEHVSKDV
Sb04g005160   VSHSEPFMN--GKNYLPGRSIVHTT--------AQKMLEPSFGQPGECFALKGSSGFVKVKLRTGIIPEAVTLEHVDKSV
OsSad1        VKHSEAFRK--AKSFMPSRNSLLEQ--------AKKMLEPSFGQPGECFALQGSSGYVEIKLRTGIIPEAVSLEHVDKSV
Os01g0267600  VRHSDAYDAGKRGGFFSSLLSGDTA------ASPKKILQPSFGEPGQCFPLQGSSGFVEIKLRKGIVPDAITLEHVSKDV
AtSUN1        MEHSDPYLVGKGSSWFATTMRRAHT-------NAVKMLSPSFGEPGQCFPLKGSEGYVQIRLRGPIIPEAFTLEHVAKSV
AtSUN2        MGHSDPFLVGNGRNWFGTSRRRVHS-------KAVKMLTPSFGEPGQCFPLKGSNGYVLVRLRAPIIPEAVTLEHVSKAV
XP_001758231  VDHSEGVFLGRGQQWSSLMFGHIVPGGTRKHPLAQKVLQPSFGQPGECLPLRGSNVFLEISLRTAIRPDAVTLEHVAKSV
XP_001776531  VDHSEGVFLGRGRQLFSMVFSSILTGEARKHPLAQKVLEPSFGEPGECLPLKGSNVFVEISLRTAILPDAVTLEHVSKSV
consensus     . **..........          .        . *.*.****.**.*....** ......*...*.*.*..**** *.*
              .......330.......340.......350.......360.......370.......380.......390.......400

ZmSUN1        AYDRSSAPKDFQVRGWYQGPHDDSEKDSNV---MSTLGEFSYDLD-KSNAQTFQLERTAQSR-VVNMVQLDISSNHGNLE
ZmSUN2        AYDRSTAPKGCRVYGWYEETPGETQSGHAA---KMALAEFAYDLE-KNNVQTFDVTAPDVG--VINMVRLDFTSNHGSSQ
Sb04g005160   AYDRSSAPKDFQVRGWYQGSHDDSEKHSNV---MAALGEFSYDLD-KSNAQTFQLERTADSR-VVNMVQLDFSSNHGNLE
OsSad1        AYDRSSAPKDFQVSGWYEGPEDDSDKESRV---VTNLGEFSYDLE-KNNAQTFQLERTADSR-VINMVRLDFCSNHGNSE
Os01g0267600  AYDMSTAPKDCRVSGWYQEAHNEAYSGHAASAKMYVLTEFTYDLD-KKNVQTFDITAPDVG--IINMVRLDFTSNHGSSA
AtSUN1        AYDRSSAPKDCRVSGSLQGPESSAETENMQ-----LLTEFTYDLD-RSNAQTFNILESSSSG-LIDTVRLDFTSNHGSDS
AtSUN2        AYDRSSAPKDCRVSGWLG--DIDMETETMP-----LLTEFSYDLD-RSNAQTFDIADSAHSG-LVNTVRLDFNSNHGSSS
XP_001758231  AYDLSSAPKEFQLYGWREMRPTDGIAQPSP--EHKLLGQFIYNSDGPSNVQTFQLSKDDVGDEPINMVRLQVLSNHGSPL
XP_001776531  AYDLSSAPKEFQLYGWREARPADRSAQPSP--VHRLLGQFVYKTDGPSNIQTFHLSKEDVGDEPINMVRLHVLSNYGSTL
consensus     ***.*.***..... *.. .   . .        .*..*.*......*.***..............*.*...**.*...
              .......410.......420.......430.......440.......450.......460.......470.......480

ZmSUN1        LTCIYRFRVHGRKPVSPSTGG------------
ZmSUN2        LTCIYRLRVHGHEPVSPGSSAGSQA--------
Sb04g005160   LTCIYRFRVHGREPGSLNTGA------------
OsSad1        LTCIYRFRVHGREPGSP----------------
Os01g0267600  LTCIYRIRVHGHEPVSPGMSVSQS---------
AtSUN1        HTCIYRFRVHGRAPDPVPVVGTNLDQDSSPESE
AtSUN2        HTCIYRFRVHGRELDSVSVAHA-----------
XP_001758231  HTCIYRIRVHGVDPQATL---------------
XP_001776531  HTCIYRVRVHGVDPQGTL---------------
consensus     .*****.****.....
              .......490.......500.......510...
```
